# Evolutionary Analysis of Snf1-Related Protein Kinase2 (SnRK2) and Calcium Sensor (SCS) Gene Lineages, and Dimerization of Rice Homologs, Suggest Deep Biochemical Conservation across Angiosperms

**DOI:** 10.3389/fpls.2017.00395

**Published:** 2017-04-05

**Authors:** Lynn D. Holappa, Pamela C. Ronald, Elena M. Kramer

**Affiliations:** ^1^Organismic and Evolutionary Biology, Harvard UniversityCambridge, MA, USA; ^2^Plant Pathology and the Genome Center, University of California DavisDavis, CA, USA

**Keywords:** abscisic acid, stress, protein kinase, SnRK2, rice, calcium, evolution

## Abstract

Members of the sucrose non-fermenting related kinase Group2 (SnRK2) subclasses are implicated in both direct and indirect abscisic acid (ABA) response pathways. We have used phylogenetic, biochemical, and transient *in vivo* approaches to examine interactions between *Triticum tauschii* protein kinase 1 (TtPK1) and an interacting protein, *Oryza sativa* SnRK2-calcium sensor (OsSCS1). Given that TtPK1 has 100% identity with its rice ortholog, osmotic stress/ABA-activated protein kinase (OsSAPK2), we hypothesized that the SCS and TtPK1 interactions are present in both wheat and rice. Here, we show that SnRK2s are clearly divided into four pan-angiosperm clades with those in the traditionally defined Subclass II encompassing two distinct clades (OsSAPK1/2 and OsSAPK3), although OsSAPK3 lacks an Arabidopsis ortholog. We also show that SCSs are distinct from a second lineage, that we term SCSsister, and while both clades pre-date land plants, the SCSsister clade lacks Poales representatives. Our Y2H assays revealed that the removal of the OsSCS1 C-terminal region along with its N-terminal EF-hand abolished its interaction with the kinase. Using transient *in planta* bimolecular fluorescence complementation experiments, we demonstrate that TtPK1/OsSCS1 dimerization co-localizes with DAPI-stained nuclei and with FM4-64-stained membranes. Finally, *OsSCS1*- and *OsSAPK2*-hybridizing transcripts co-accumulate in shoots/coleoptile of drying seedlings, consistent with up-regulated kinase transcripts of *PKABA1* and *TtPK1*. Our studies suggest that interactions between homologs of the SnRK2 and SCS lineages are broadly conserved across angiosperms and offer new directions for investigations of related proteins.

## Introduction

Environmental stress induces a wide array of plant responses in molecular pathways of stress perception and tolerance, including gene expression in seed and vegetative tissues (Sah et al., 2016). Abscisic acid (ABA) is called the plant stress hormone due to its characteristic rapid accumulation in vegetative tissues upon stress exposure (Cutler et al., 2010; Finkelstein, 2013; Sah et al., 2016). Protein kinases modulate critical response components of plant stress physiology and cell biology and are the subject of intense molecular investigation (Lehti-Shiu and Shiu, 2012). Despite this, explicit evolutionary studies of the conservation of stress and kinase response pathways are slowly emerging across diverse plant species, although work is most often conducted in grasses and *Arabidopsis*.

Sucrose non-fermenting (Snf1)-related protein kinases of Group2 (SnRK2s) are essential for both ABA-dependent and ABA-independent environmental stress-signaling responses (Todaka et al., 2015). ABA induces the expression of specific target genes via ABA-response element protein/ABRE binding factor (AREB/ABF) transcription factors. The first cereal *SnRK2* gene identified was a breadwheat protein kinase with an ABA-upregulated mRNA, called PKABA1, which transiently could suppress gibberellic acid (GA)-induced α-amylase transcriptional expression in barley aleurone (Anderberg and Walker-Simmons, 1992; Holappa and Walker-Simmons, 1995; Gómez-Cadenas et al., 1999). PKABA1 could also phosphorylate the *Triticum aestivum*, TaABF, which is the ortholog of *Arabidopsis thaliana ABI5*, a basic leucine zipper (bZIP) transcription factor (Johnson et al., 2002). In contrast, drought conditions can also induce *SnRK2s* and stress-response genes without ABA mediation via other transcription factors, including dehydration-responsive element binding protein/C-repeat binding factors, DREB/CBFs and NAM/ATAF/CUC, NACs (Singh and Laxmi, 2015). Extensive research has highlighted the contributions of SnRK2s to a diverse set of environmental, metabolic, and nutrient responses through differential expression as well as differential activation by phosphorylation in response to ABA and dehydration-type stress (Hauser et al., 2011; Fujii and Zhu, 2012; Nakashima and Yamaguchi-Shinozaki, 2013; Todaka et al., 2015; Yoshida et al., 2015).

Studies in the model plant *A. thaliana* have shown that reversible phosphorylation between SnRK2s and protein phosphatases group 2C (PP2Cs) is modulated via ABA and proteins known as PYR/PYL/RCAR proteins (Ng et al., 2011; Soon et al., 2012; Zhou et al., 2012; Finkelstein, 2013). In the absence of ABA, *A. thaliana* SnRK2s (2.2, 2.3, 2.6) remain inactive, as PP2Cs maintain the de-phosphorylated state of the kinases. In the presence of ABA, these SnRK2s remain phosphorylated and active, as PP2Cs interact with ABA-bound PYR/PYL/RCARs and can no longer de-phosphorylate the kinases. These now activated SnRK2s can phosphorylate downstream proteins, including bZIPs, to facilitate transcription of ABA-responsive genes (Sirichandra et al., 2010; Ng et al., 2011; Soon et al., 2012; Zhou et al., 2012; Finkelstein, 2013). Studies in rice have similarly identified many components of this ABA signaling pathway, including 10 SnRK2s known as osmotic stress/ABA-activated protein kinases SAPKs (Kobayashi et al., 2004), 10 clade A PP2Cs and 12 PYLs (Kim H. et al., 2012; He et al., 2014; Kim et al., 2015). Therefore, this ABA-response pathway is conserved across Arabidopsis and rice, angiosperm species representative of the two major classes of flowering plants.

Phylogenetically, SnRK2s have been grouped into a kinase superfamily known as the calcium-dependent protein kinases and Snf1-Related Kinases, or CDPK-SnRKs (Hrabak et al., 2003). A direct regulatory role for calcium has been established for members of the CDPK family because they all encode a single polypeptide with the catalytic kinase domain fused to a C-terminal calmodulin-like domain possessing four signature calcium-binding EF-hand sites (Harmon, 2003; Harper and Harmon, 2005). Functionally, Ca^2+^ has been shown to induce conformational changes to promote kinase phosphorylation activity (Harper and Harmon, 2005; Schulz et al., 2013). In contrast, the SnRK kinases all lack EF-hand calcium motifs and fall into three distinct subfamilies, designated SnRK1, SnRK2, and SnRK3, based on variation in their N- and C-terminal sequences (Harmon, 2003; Hrabak et al., 2003; Kulik et al., 2011). SnRK1, the only clade with homologs in yeast and mammals, appears to function independently of Ca^2+^, although as part of a multi-protein complex (Avila et al., 2012; Broeckx et al., 2016). However, calcium is required for the activity of SnRK3s, also known as the calcineurin B-like protein interacting kinase, CIPKs (Luan et al., 2009; Kleist et al., 2014; Edel and Kudla, 2015).

Despite the common regulatory role of Ca^2+^ for CDPKs and SnRK3s, evidence for the regulation of SnRK2s by Ca^2+^ was initially limited to the implication that the acidic residues in the C-terminal domain could bind cations such as calcium (Anderberg and Walker-Simmons, 1992; Hrabak et al., 2003; Holappa et al., 2005). Recent reports have now shown that Ca^2+^ directly modulates SnRK2-mediated responses. For example, phosphorylation of AREBP BZIP proteins by heterologously expressed SnRK2 activity was enhanced by addition of CaCl_2_ to wheat root extracts (Coello et al., 2011). Recently, a *Nicotiana tabacum* SnRK2 (NtOSAK) was used as bait to screen a yeast two-hybrid (Y2H) library prepared from *Nicotiana plumbaginifolia* cDNAs (Bucholc et al., 2011). The recovered interacting protein was called *N. plumbaginifolia* SnRK2-interacting Calcium Sensor (NpSCS) due to its signature EF-hand sequence motifs and its ability to bind Ca^2+^ *in vitro*. However, even though *E. coli* produced recombinant NtOSAK could phosphorylate NpSCS *in vitro*, its activity was unaffected by CaCl_2_ or EGTA. Similar experiments confirmed that three *A. thaliana* SnRK2s (2.4, 2.6, 2.8) can dimerize with and *in vitro* phosphorylate a respective homolog AtSCS; but in this case, addition of CaCl_2_ reduced the phosphorylation activities (Bucholc et al., 2011). In rice, two SCS homologs (Os03g14590, Os10g09850) were identified from a comprehensive screen for kinome interactors using rice SnRK2s, also called *Oryza sativa* osmotic stress/ABA-activated protein kinases, OsSAPK1-7 (Ding et al., 2009; Seo et al., 2011), thus further implicating calcium in stress responses.

Given the essential roles of SnRK2s and their calcium sensor partners in ABA responses, we wanted to broaden the knowledge of SCSs beyond the eudicot models of *Arabidopsis* and *Nicotiana*. To this end, we conducted extensive phylogenetic analyses of both the kinases and their calcium sensor homologs to better understand their evolutionary relationships. As *Triticum tauschii* protein kinase 1 (TtPK1) hybridizing transcripts were highly abundant in shoots of ABA-elevated, dehydrated leaves (Holappa et al., 2005), we sought to identify TtPK1-interacting partners in leaves during stressed seedling establishment. Here, we report the recovery of *Oryza sativa* SnRK2-calcium sensor (OsSCS1) from our screen of rice seedling Y2H libraries using TtPK1 as bait. We also show that removal of its C-terminal end along with its N-terminal EF-hand abolishes its interaction with the kinase. Our fluorescent protein fusion analyses revealed that OsSCS1 alone and its dimer with TtPK1/OsSAPK2 have nuclear and cytoplasmic distributions, implicating these sites for their regulatory functions. Our results, taken together with *Nicotiana* and *Arabidopsis* SCS orthologs, suggest that SCSs are likely to modulate SnRK2 interactions across diverse species.

## Materials and methods

### Gene identification and phylogenetic analyses

We obtained predicted protein sequences from public databases, by BLAST searching of online databases, including NCBI, Phytozome (Goodstein et al., 2012), DFCI (Antonescu et al., 2010), 1000 plants (Matasci et al., 2014), and Ensembl genomes (Kersey et al., 2016). In some cases, available genome sequences were combined with EST data to extend and correct predicted protein sequences. Redundant sequences (99% identity or more) from the same organism were not included. Regions that could not be confidently aligned across the entire dataset were excluded from the analysis. Gene accession information is provided in Supplementary Tables 2–5. For dataset 1, multiple sequences were initially aligned using ClustalW followed by manual adjustment in MacVector 12.7 and Aliview1.4 (Larsson, 2014). For dataset 2–4, multiple sequences for each dataset were aligned as implemented by MAFFT, or PASTA. The motif alignment and search tool, MEME/MAST (Bailey et al., 2009) was used on dataset 3. See Supplementary FASTA Files for this multiple sequence alignments (Posada and Crandall, 1998; Posada and Buckley, 2004). All phylogenetic analyses were conducted with the maximum likelihood (ML) method as implemented by RAxML 7.2.6 via the CIPRES Science Gateway V.3.1 (http://www.phylo.org/sub_sections/portal/). The GTR+G model was selected by ModelTest 3.07, as well as per the suggestion of the RAxML manual (Stamatakis, 2006). Each analysis was run with 1,000 bootstrap replicates using the CIPRES Science Gateway V.3.1 (http://www.phylo.org/sub_sections/portal/).

### Yeast two-hybrid analyses

We screened yeast two-hybrid (Y2H) libraries were comprised of three rice cDNA libraries prepared from 14 to 21 days old rice seedlings that were exposed to abiotic and biotic treatments as described (Seo et al., 2011), using the *T. tauschii* ortholog to PKABA1 of bread wheat, TtPK1 (Holappa et al., 2005). This Y2H screen was conducted in parallel to a larger rice kinome screen (Ding et al., 2009; Seo et al., 2011). The full-length coding sequence of *TtPK1* (GenBank AY714526) was PCR-amplified from the original pBluescript clone using primers (Supplementary Table 1) cloned into pENTR-D-topo vector using topoisomerase following manufacturer's protocol (Invitrogen, Carlsbad, CA). A recombination reaction was conducted using the Gateway LR Clonase Enzyme Mix (Invitrogen, Carlsbad, CA), *TtPK1*-pENTRD-topo entry vector, and the pBD destination vector. The resulting *TtPK1* “bait” vector in pXDGATcy86 (a derivative of pMC86) was confirmed by sequence analysis to be the GAL4 DNA-binding domain fused in-frame to the N-terminal methionine codon of *TtPK1*. The vectors were modified as described in Ding et al. (2009) and Seo et al. (2011) with the bait screening vector a derivative of pXDGATcy86-GAL4-BD and target vectors pAD-GAL4-2.1.

#### Autoactivation pre-screen

The TtPK1 bait construct was transformed into the MATa yeast strain HF7c and subjected to testing for bait autoactivation of the His3 reporter. TtPK1 had some low level autoactivation growth, but was reduced or completely eliminated by the addition of 2.5 mM 3-amino-1,2,4-triazole medium (3-AT). These levels of 3-AT were used to screen the Y2H cDNA libraries.

#### Mating screen

HF7c cells containing the bait BD-TtPK1 fusion in pXDGATcy86 were mated with the Y187 (MATα) cells containing the Y2H AD-fusions to cDNA libraries. Cultures (bait MATa and prey MAT α) were grown to densities of ~108 to 109 colony forming units (CFU)/ml. An ~2:1 ratio of “bait” to target haploid cultures were mixed in 20 ml YPAD and incubate 20–24 h. Mating was induced with low pH YPAD and monitored using microscope. Resultant mating mixture was filtered through 0.45 μm filter, spread onto YPAD plates pH 4.5, incubated 5–7 days on selective media, and monitored for His+ and LacZ+ yeast colonies. Positive colonies were isolated and washed with sterile water, and then transferred to synthetic dropout (SD) media plates. After ~10 days of yeast growth, the plasmids were rescued by rolling circle amplification (RCA) from the yeast colonies grown on selective medium (Ding et al., 2007). Each yeast colony was transferred to new plate with selection by “patched” method and allowed to grow to increase biomass. To recover plasmids, minipreps were conducted on each yeast patch using Zymolase (Zymo Research, Irvine, CA) to initially solubilize cell walls and then standard alkaline lysis protocol. The low-copy number plasmids were then transformed by electroporation into highly competent *E. coli* and selected on ampicillin for recovery of pAD. Plasmids with putative TtPK1-interacting proteins were sequenced.

#### Autoactivation post-screen

Each AD or BD construct was individually transformed into AH109 to test for auto-activation. No auto-activation occurred with the AD vectors on SD media lacking histidine and leucine (−HL). Autoactivation did occur when AH109 contained the BD construct with full-length TtPK1 or PKABA1, but growth was minimized with the addition of 20 mM 3-AT to on SD media lacking histidine and tryptophan (−HT) media plates. Autoactivation also did occur when AH109 contained the BD construct with OsSCS1, but was minimized with the addition of 3-AT.

#### Y2H assays

Coding regions of *TtPK1* and *PKABA1* were PCR-amplified with the addition of four nucleotides (cacc) to the 5′ end for cloning into the Gateway entry vector, pENTR-D-topo (Invitrogen). A catalytically-dead TtPK1 lacking the GSGNFG nucleotide binding site was prepared using a modified 5′ end primer without the codons and a 3′ stop codon primer (Supplementary Table 1). For the other Y2H constructs (**Figure 5**, Supplementary Figures 4, 5), coding regions of *TtPK1, 14SSPSE*, and/or *12SSPSE* were PCR-modified with 5′ and 3′ flanking *Eco*RI sites and cloned into pCR4-topo using topoisomerase (Invitrogen). The subsequent inserts of 14LIE, 12LIE, 14QPASQD, 12QPASQD (or 12Δ, Supplementary Figure 6) were released with *Eco*RI and then cloned in-frame into pGBKT7 or pGADT7 vectors (Clontech Mountain View, CA). Indicated Y2H protein pair combinations (Supplementary Figures 4C, 5C) of bait TtPK1 and prey OsSCS1 in screening vectors were sequentially transformed into yeast host strain AH109. Manufacturer's positive (p53/T-antigen) and negative (empty/CL1, LAM/empty) controls were used to monitor Y2H assays. Two *Aquilegia vulgaris* MADS proteins (AqAP31, AqPI), known for their “strong” protein-protein interaction (Kramer et al., 2007), were also used as an additional Y2H positive control. All Y2H experiments were conducted in triplicate, as described in Kramer et al. (2007), and manufacturer (Clontech Mountain View, CA) with the following modifications. Host AH109 colonies transformed with paired Y2H constructs, as indicated, were grown for 3 days at 30°C on fresh non-restrictive (LT) plates. For each assay, a single colony (~2 mm diameter) transformed with paired Y2H constructs was used to innoculate 4 mL of 0HLT liquid media. After 18 h incubation (shaking at 30°C), each culture was diluted to OD600 of 0.5 and this same diluted culture was used for both the liquid and plating Y2H assays. For the liquid assay, an aliquot of diluted culture (OD600 of 0.5) was monitored for secreted α-galactosidase into the media after 18 h growth. The activity assay measured the accumulation of soluble yellow product after 1 h at 410 nm after the addition of substrate, *p*-nitrophenyl α-D-galactopyranoside (Sigma N0877) to an aliquot of diluted culture. For the plate assay, two microliters of this same culture (OD600 of 0.5), along with two 10-fold serial dilutions, were pipetted onto selective or restrictive plates and grown for 4 days. These dilutions are designated 0, −1, and −2 for 10^0^, 10^−1^, and 10^−2^ (top of middle panel, Supplementary Figures 4C–E, 5C).

### Co-immunoprecipitation (Co-Ip)

Constructs in Supplementary Figure 6 were prepared with versions of coding regions of *TtPK1, 14SSPSE*, and/or *12SSPSE* were modified by PCR using primers with 5′- and 3′-flanking *Eco*RI sites and cloned with topoisomerase into pCR4-topo vector (Invitrogen). Subsequently, *Eco*RI-released inserts were ligated in-frame to N-terminal tags of pET-28a^+^ or pCITE-4b^+^ (Novagen EMDMillipore). The modified plasmids containing 14SSPSE, 12SSPSE, and 12QPASQD (or 12Δ) were linearized with *Sph*I for pET28a^+^ or *Sca*I for pCITE-4b^+^ and ethanol-precipitated with 3 M sodium acetate. The TNT Coupled Reticulocyte Lysate System (RRL) (Promega, Madison, WI) was used to express the recombinant proteins from the linearized plasmids in independent *in vitro* reactions. We conducted our experiments following manufacturer's protocols with modifications described in detail by Wang et al. (2002). In our experiments, N-terminal T7 peptide tags fused in-frame to coding regions of 14SSPSE, 12SSPSE, or 12Δ (including amino acids 103–226 of OsSCS1) were prepared by cloning into pET-28a^+^. The full coding region of TtPK1 was cloned in-frame into pCITE-4b^+^. Components for the non-radioactive (“cold”) RRL reactions were assembled to express the T7-tagged pET28a proteins and the empty vector in separate RRL reactions. An aliquot from each reaction was analyzed with poly-acrylimide gel electorphoresis (PAGE) to confirm the quality and length of polypeptides. In separate radioactive reactions, the empty vector or TtPK in pCITE4a^+^ were assembled with RRL components and the addition of ^35^S methionine (NEN/Amersham). Each RRL reaction was stopped by the addition of cyclohexamide after ~120 min.

For the co-Ip reactions, we used T7-tag antibody Protein A-Sepharose (Amersham Pharmacia Biotech), which was washed and blocked buffer with Washing buffer-100 (W100: 20 mM Tris OAc, pH 7.5, 10% glycerol, 1 mM EDTA, 5 mM MgCl_2_, 0.2 M NaCl, 0.1 M KGlu, 1% NP40, 0.5 mM NaDeoxycholate, 1 mM DTT) containing 0.5 mg/ml lysozyme, 0.5 mg/ml BSA, 0.05 mg/ml glycogen, 1 mM DTT. Briefly, for our reactions, we mixed 60 μl ^35^S-labeled TtPK-pCITE (“hot”) or empty pCITE with 20 μl T7-tagged (“cold”) 14SSPSE, 12SSPSE, or 12Δ or empty pET28a (pET, Supplementary Figure 6). Each paired protein reaction was placed in a tube with 240 μl of W100 rocked at 4°C for ~1 h, and centrifuged for 15 min at 160 × g. An aliquot of the supernatant from each paired-protein reaction was mixed with T7-tag antibody Protein A-Sepharose (washed, blocked) and rocked at 4°C for ~2 h. Each paired-protein reaction was then washed with W300 buffer [W100 buffer plus 200 mM KGlu (with final concentration of KGlu 300 mM) and 1 mM DTT] and pelleted by centrifugation. This wash step was repeated twice. The paired-protein reaction was then placed in 30 μL TMG buffer (10 mM TrisOAc pH 8.0 1 mM MgCl_2_ 10% Glycerol 1 mM DTT). Each reaction was boiled for 3 min, spun down, and loaded onto a prepared PAGE gel (BioRad, Richmond CA) for size fractionation. PAGE gels were vacuum-dried (LabConco, Kansas City, MO) onto filter paper, exposed to a phosphor-imaging screen for 3–10 days of exposure, and visualized by laser densitometry and ImageQuant software (Molecular Dynamics, Sunnyvale, CA) in an imaging facility at Harvard University. Total protein (5–10 μg/5 μl) of was mixed with Laemmli buffer (20 mM Tris, 1% SDS, 0.05% bromphenol blue, 10% glycerol, pH 6.8) and incubated at 96°C for 5 min. The samples were resolved on at 12 or 15% SDS-containing polyacrylamide gels, either manually or commercially prepared (BioRad, Richmond CA). The electrophoresis was conducted at a constant current of 20 mA. Where necessary, proteins were transferred onto PVDF membrane (BioRad, Richmond CA) with a semi-dry blotting device following Manufacturer's manual (BioRad, Richmond CA).

### Bimolecular fluorescence complementation and subcellular localization assays

To complete the putative full-length *OsSCS1* coding region, we added 153 nucleotides to the 5′ end of the *14SSPSE* clone. Successive rounds of PCR were conducted to extend *14SSPSE* cDNA inserts, using the 5′ primers listed in Supplementary Table 1 and the 3′ stop codon primer. Due to the high GC-nature of the 5′ sequences, *Taq* polymerase plus Q buffer (Qiagen, Valencia, CA) was used to stablilize the amplicon and minimize “slippage” during the PCR. Resulting PCR products were cloned by topoisomerase into pCR4-topo vector and sequenced to confirm.

For construct preparation, versions of *TtPK1* and *OsSCS1* were PCR-modified with primers (Supplementary Table 1) and cloned with topoisomerase into the Gateway entry vector, pENTR-D-topo (Invitrogen). Each entry vector insert was transferred to a destination vector, using LR clonase and manufacturer's protocol (Invitrogen, Carlsbad, CA). Each destination Gateway vector (a single binary plasmid prepared in the pGREEN-II backbone vector) and the helper plasmid pSOUP were gifts from Dr. Detlef Weigel at the Max Planck Institute, Tubingen, Germany. The fluorescent protein (FP) destination vectors were N-terminal green (G)FP (pFK241), C-terminal GFP (pFK242), C-terminal red (R)FP (tomato pJV110), N-terminal split-*m*citrine yellow (Y)FP (pAS54, pAS56), and C-terminal split-*m*citrine YFP (pAS58). The 3′ends of versions of *TtPK1* and *OsSCS1* were PCR-modified to remove the stop codon for the in-frame C-terminal FP fusions.

Each recombinant *Agrobacterium tumefaciens* strain containing a destination Gateway vector was selected on plates containing LB media [25 μg/mL rifampicin, 50 μg/mL gentamycin, 5 μg/mL tetracycline, 100 μg/mL spectinomycin (RGTS) at 28°C]. Four colonies were placed in 5 mL LB media with RGTS and grown overnight shaking at 28°C. *Agrobacteria* cultures were centrifuged to remove media and bacteria were then adjusted to an OD600 of 0.1 in 50 mM MES buffer, pH 5.7. Acetosyringone (Sigma-Aldrich) was added to a final concentration of 150 μM and cultures were placed at room temperature for ~2 h. *Nicotiana benthamiana* seeds (a gift from Dr. Thomas Brutnell, Danforth Plant Science Center) were germinated in potting soil in trays in a greenhouse in light with temperature at ~25–30°C. Seedlings were transferred to pots and grown, well-hydrated, until ~3–4 leaf stage. *A. tumefaciens* strain GV3101 was transformed via electroporation using protocol described in Weigel and Glazebrook (2006).

*Agrobacterium*-infiltration protocols were modified based on details described in Goodin et al. (2002, 2007) and Kanneganti et al. (2007). One milliliter of the *Agrobacterium* suspension buffer at OD600 of 0.1 was infiltrated into an abaxial section of a fully expanded leaf. For co-infiltration, equal volumes of two suspensions at OD600 of 0.2 were combined and then infiltrated. All infiltrations were conducted using a syringe without needle on the lower side of a transiently transformed leaf. After 1–7 days post-infiltration, plants to be imaged were kept in the dark next to microscope. An abaxial leaf section (~2 cm^2^) was mounted with water on a glass slide with 1.5 coverslip and W-solution. Infiltrated sections of leaves were visualized and imaged at room temperature by a Zeiss inverted confocal laser scanning microscopy LSM510 with argon and ZEN software (Zeiss) in the Biological Imaging Center at Harvard University. To visualize DNA-containing organelles, leaves were infiltrated with a DAPI (4′,6-diamidino-2-phenylindole) solution at 1 μg/mL DAPI solution (1 μg/mL). To visualize the plasma membrane and endocytotic membranes (Bolte et al., 2004), leaves were infiltrated with a 50 μM FM4-64 (Invitrogen) solution ~0–2 h prior to imaging.

GFP and YFP were excited at 488 nm and the emitted light captured at 505–555 nm; light emitted at 630–680 nm recorded chlorophyll autofluorescence. RFP (tomato) was excited using 543 nm and captured at 590–630 nm. Light emitted at 630–680 nm recorded chlorophyll autofluorescence. The detection settings were chosen according to the fluorophores. Excitation of GFP and YFP with argon laser was at 488 nm, of mRFP, FM4-64, and mCherry with helium-neon laser at 543 nm.

### Plant material and northerns

Whole rice grains were germinated in covered Petri dishes with water in a growth chamber in the darkness at 30°C. At 2 days covers were removed from Petri dishes so seedlings now had light. To maintain fully hydrated conditions, all seedlings in petri dishes were grown so roots were bathed in water in growth chamber at 30°C with a 16-h photoperiod (100 μE/m^2^/s) at 100% RH. The Petri dishes were placed inside a plastic bin with a plastic wrap cover. Seedlings were grown for ~14 days. For each treatment, 30 whole seedlings were placed in a Petri dish with or without water for 16 h (Supplementary Figure 9B) or 0, 2, 4, 8 h (Supplementary Figure 9C). Experiments for northern analyses were conducted in triplicate.

Total RNA extraction, size-fractionation, northern transfer, and hybridization conditions were as reported in Holappa and Walker-Simmons (1995). For these studies, ~2.5 mg total RNA was loaded per lane. Nylon membranes (0.45 μM, Whatman Nytran SPC) were exposed to phosphor-imager screen and analyzed by laser densitometry with ImageQuant software (Molecular Dynamics, Sunnyvale, CA). PCR-derived products spanning the coding region of the *14SSPSE* cDNA (705 bp) or the *TtPK1* cDNA (1161 bp) were used as probes, labeled with ^32^P by random priming (Stratagene, La Jolla, CA, USA). An rDNA ^32^P end-labeled 26 base oligonucleotide, were used to assess RNA quality and quantity.

## Results

### Patterns of SnRK2 and OsSCS1 evolution

To obtain a broad overview of the conservation of both the SnRK2 and SCS gene lineages, we performed detailed phylogenetic analyses of the two gene families. Our studies considered four distinct datasets: (1) the superfamily of SNF1-related plant kinases, with 246 taxa; (2) the SnRK2 family and its relationship to the pan-eukaryotic SnRK1s, with 285 taxa; (3) the SCS lineage with its most closely related calcium EF-hand proteins, with 67 taxa; and (4) a detailed analysis of the SCS lineage *sensu strictu* together with what we have termed the SCSsister lineage, with 231 taxa.

### The SnRK2 phylogeny

To better understand comparative patterns of evolution between the SnRK2 lineage and its partner SCS homologs, we assembled a superfamily matrix termed dataset1 containing broadly sampled representatives of all three major SNF1-like lineages: the SnRK1s, SnRK2s, and SnRK3s. This phylogeny, which was based only on domains conserved across the entire superfamily, demonstrates considerable differentiation among the separate clades (Supplementary Figure 1).

The SnRK1 family exhibits few internal duplication events and is notable for its extremely short branch lengths, indicating a high level of sequence conservation. We observed that all of the included fungal and animal sequences are tightly associated with this clade. These results suggest that the divergence between the ancestor of the SnRK1 lineage and that of the SnRK2+3 lineage occurred before the split between these major eukaryotic kingdoms. In contrast to SnRK1, the SnRK3 family is marked by a high degree of sequence differentiation with strong support for many internal sublineages and longer branch lengths. By comparison, the SnRK2 family shows an intermediate degree of diversification and fewer internally supported subfamilies. The lack of definition within the SnRK2 clade reflects the fact that the regions used for the superfamily analysis were not sufficient to distinguish among SnRK2 duplication events.

**Figure 1:**
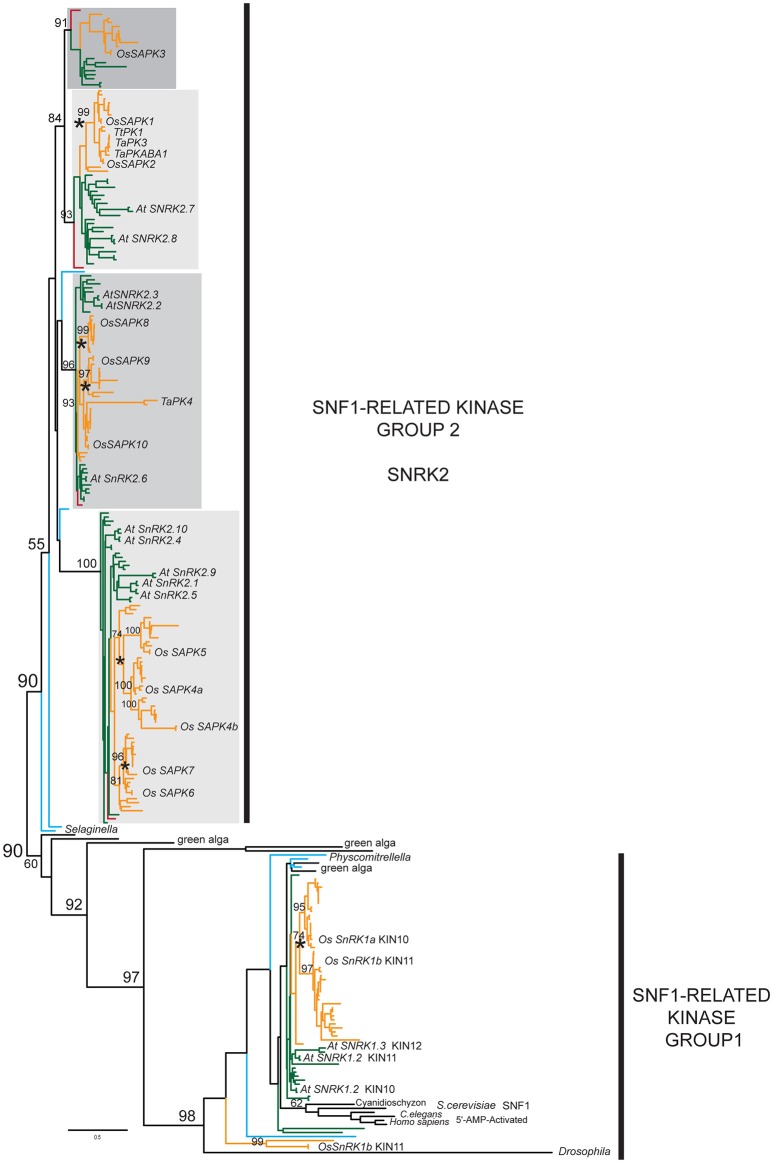
**Simplified SnRK2 phylogeny**. Maximum likelihood (ML) phylogeny of SnRK2 members inferred from 285 taxa and 335 amino acid characters, rooted relative to the SnRK1/SNF1/AMPK clade. The detailed tree is presented in Supplementary Figures 2A–D. ML bootstrap values above 50% are shown above key clade branches. Orange, green, red, and teal colored branches denote monocot, dicot, *Amborella*, and *Selaginella moellendorfi* and *Physcomitrella patens* representatives, respectively. Four distinct, highly supported (>90%) SnRK2 clades are identified by gray rectangles. Rice and Arabidopsis sequences are labeled in bold text with the first letter of genus and species and protein accession numbers. Asterisks denote ploidy duplication events. The list of taxa and their multiple sequence alignment used in this phylogeny are provided in Supplementary Table 3 and Supplementary File 2 in Phylip data format.

To further explore SnRK2 evolution, we constructed dataset2 using larger portions of the protein coding sequence. We took advantage of sequences from the rapidly growing genomic and transcriptomic databases to reconstruct a more detailed SnRK2 phylogeny using the SnRK1 clade as an outgroup. Additionally, we utilized the PASTA algorithm to generate “unbiased” non-manual multiple sequence alignments, which were analyzed with the ML method. In Figure 1, we show a simplified version of a detailed kinase phylogeny (Supplementary Figures 2A–D) that demonstrates considerable differentiation among distinct SnRK2 lineages, corresponding to the OsSAPK3, OsSAPK1-2, OsSAPK8-10, and OsSAPK4-7 clades. Note that several different naming systems have been used in past phylogenetic analyses of SnRK2 so we will simply refer to them based on their rice representatives.

The SnRK2 tree has four major clades of seed plant members, each with previously recognized model system representatives. These strongly supported clades include gymnosperm homologs in all cases but the lycopod and moss SnRK2 homologs cannot be associated with any of them with high confidence. This clearly demonstrates that the four classes of SnRK2 were established before the radiation of the seed plants but we cannot determine the timing of the duplication events without more non-seed plant data. Unlike the SnRK1 clade, which contains green alga representatives, our search did not uncover any pre-land plant SnRK2 homologs.

Previous studies have treated the OsSAPK3 clade together with the closely related OsSAPK1-2, but as each clade clearly contains homologs from across the angiosperms, we believe it is appropriate to define them separately. We identified orthologs in many dicots, including legumes, *Nicotiana* and *Aquilegia*, but we found no Arabidopsis SAPK3 ortholog. Furthermore, the OsSAPK3 homologs appear to have weaker conservation of the C-terminal ABA box raising questions as to whether they function similarly (Kobayashi et al., 2004).

We also found evidence for subsequent duplications within each of the subfamilies, particularly in the ancestor of grasses that gave rise to the separate OsSAPK1 and 2 lineages in the OsSAPK1-2 clade, and the separate OsSAPK8, 9, and 10 lineages in the OsSAPK8-10 clade. In the OsSAPK4-7 clade, two rounds of pre-Poales duplications appear to have occurred, giving rise to four separate lineages defined by OsSAPK4-7. Likewise, duplications have also occurred within the core eudicots, possibly in conjunction with the gamma hexaploidization event that marks the base of the core eudicots (Jiao et al., 2012). These are seen in the OsSAPK1-2 clade, as represented by the Arabidopsis homologs AtSnRK2.7 and AtSnRK2.8; the OsSAPK8-10 clade, represented by AtSnRK2.2/2.3 and AtSnRK2.6; and the OsSAPK4-7 clade, represented by AtSnRK2.1/2.5/2.9 and AtSnRK2.4/2.5. The later duplications seen in Arabidopsis (e.g., AtSnRK2.2 and AtSNRK2.3) are likely due to Brassicales-specific α- or β-genome duplications (Vision et al., 2000). It is also notable that while most SnRK2 sequences exhibit moderate branch lengths, a few are associated with extremely long branch-lengths that possibly indicate diversifying selection, particularly TaPK4 from wheat.

Each SnRK2 lineage is associated with sequence synapomorphies (i.e., shared, derived features). For instance, the OsSAPK8-10 clade displays N-terminal extensions not observed in the other lineages. Although the C-terminal SnRK2 box (Ng et al., 2011) is well-conserved across all homologs, the ABA box shows variable degrees of conservation with glutamic acids dominating in the OsSAPK4-7 clade while the OsSAPK3 clade appears to have the lowest conservation.

### SCS phylogeny

We next investigated the evolution of SnRK2-calcium sensor proteins (SCSs). The first step in this process was to identify closely related lineages, for which we referred to Day et al. (2002), which identified AtSCS (At4g38810) as related to four other proteins (At1g54530, At2g44310, At5g28830, and At5g22760). When we examined these five proteins in more detail, we observed that only the EF-hand domains were conserved and that At5g22760 encodes a PHD finger family protein that appears to share little overall similarity to the others. We, therefore, carried out an unrooted analysis of homologs of the remaining four genes (At4g38810, At1g54530, At2g44310, and At5g28830). Along with sequences from the model species Arabidopsis and rice, we also incorporated a range of representatives from select eudicots, monocots, the lycophyte *S. moellendorffii*, and the moss *Physcomitrella patens*.

**Figure 2:**
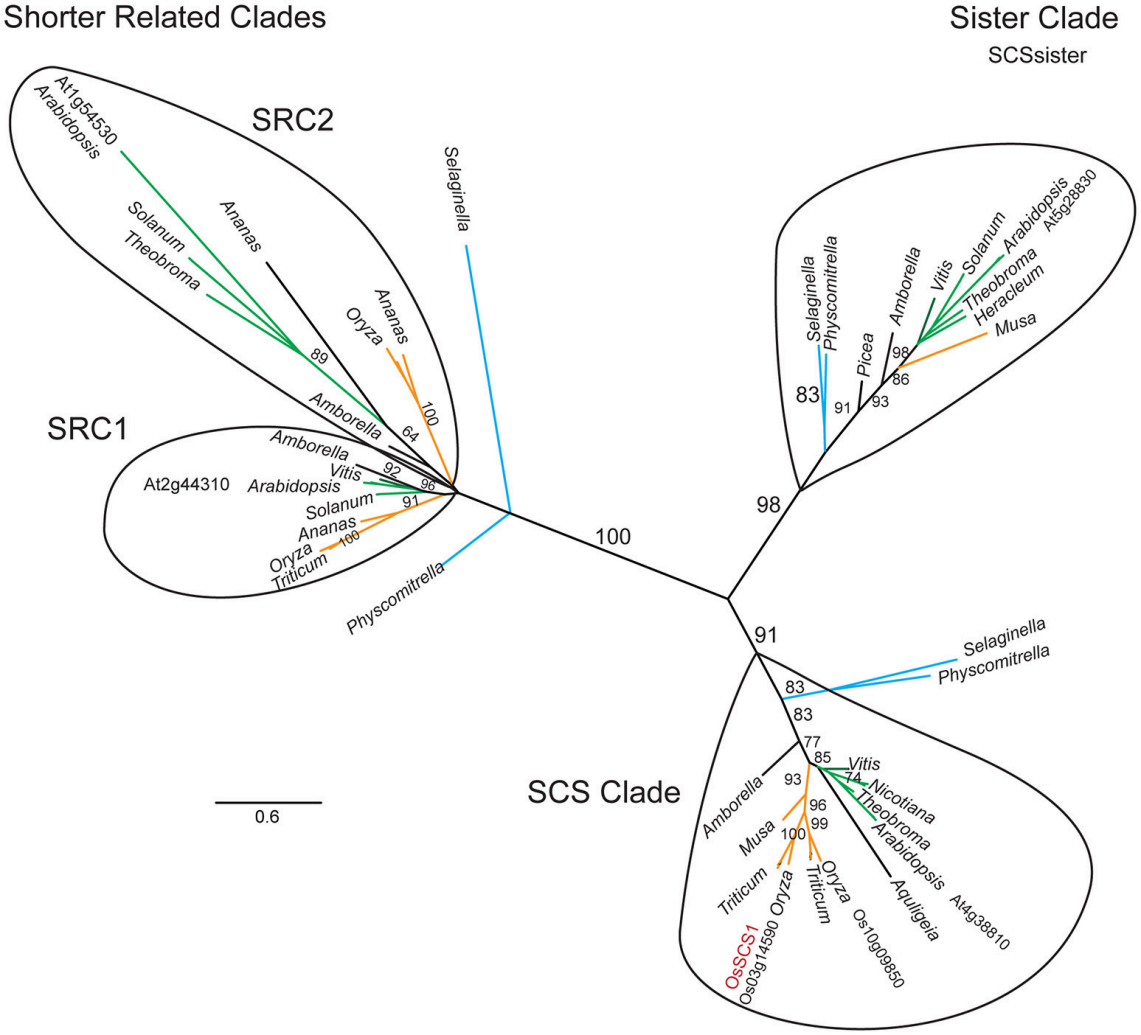
**SCS1 unrooted phylogeny**. Maximum likelihood (ML) phylogeny of OsSCS1, sister (SCSsister), and shorter related clades (SRC1 and 2) inferred from 67 taxa and 863 amino acid characters. ML bootstrap values above 70% are shown above key clade branches. Orange, green, and teal colored branches denote monocot, dicot, and *Selaginella moellendorfi* and *Physcomitrella patens* representatives, respectively. First letter of Genus and species and protein accession numbers are provided for Arabidopsis, rice, and wheat sequences. Four distinct clades are circled. The Genera is provided for other taxa. The list of taxa and their multiple sequence alignment used in this phylogeny are provided in Supplementary Table 4 and Supplementary File 3 in Phylip data format.

**Figure 3:**
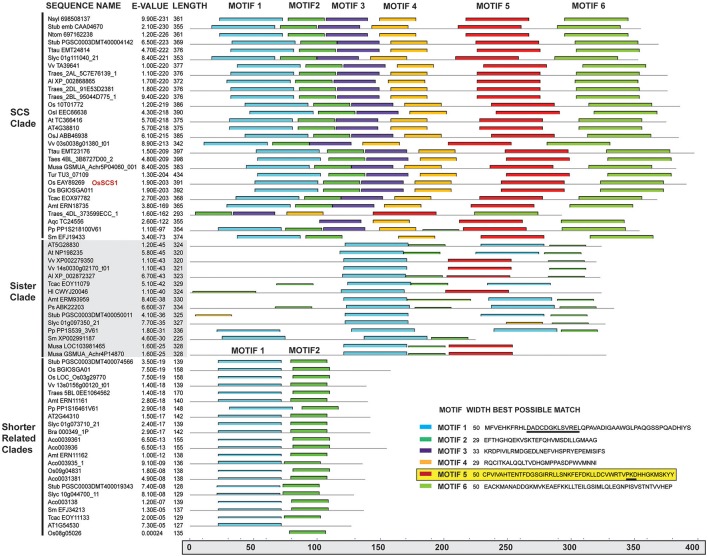
**MEME/MAST motif analysis of OsSCS1 and related clades**. Columns (left to right) show clades, sequence names, *E*-values, amino acid lengths, and conserved motifs for the 67 taxa from the unrooted tree (Figure 2) identified by MEME/MAST analysis. A key for the conserved motifs 1–6 is shown in lower right corner. Each colored bar represents the amino acid sequence for each motif and where it is located within the schematic of the above sequence(s). The lower gray bar indicates the amino acid position from 0 to 400 residues. Underlined in motif 1 is the N-terminal Ca^2+^ EF hand motif. Highlighted motif 5 is region needed for Y2H interaction. The list of taxa used in this phylogeny is provided in Supplementary Table 4.

Our phylogenetic analysis of dataset3 revealed four distinct clades, each corresponding to one Arabidopsis representative (Figure 2). The unrooted tree shows that each lineage was established before the origin of angiosperms, as they all contain an *Amborella* homolog. They appear to fall into two pairs: the SCS clade itself and what we call the SCS Sister Clade (SCSsister), and two clades that we refer to collectively as the Shorter Related Clades (SRC1 and 2). The SCS and SCSsister clades each contain homologs from non-seed vascular (*Selaginella*) and non-vascular (*Physcomitrella*) plants, suggesting an early divergence in land plant evolution. Although there are *Physcomitrella* and *Selaginella* homologs strongly associated with the two shorter related clades, these homologs pre-date the separation of the two angiosperm lineages, suggesting that the ancestor of all land plants had three homologs, one each for the SCS, SCSsister, and SRC homologs. When we ran a MEME/MAST analysis on this dataset3, we found that all four clades contain a version of a classic EF-hand sequence: DADCDGKSVREL (motif 1 in Figure 3). The SCS and SCSsister clades also share a longer stretch of C-terminal motifs (motifs 1, 5, and 6), confirming their closer relationship. The two SRCs share only two N-terminal motifs (motifs 1 and 2) with the two other clades.

**Figure 4:**
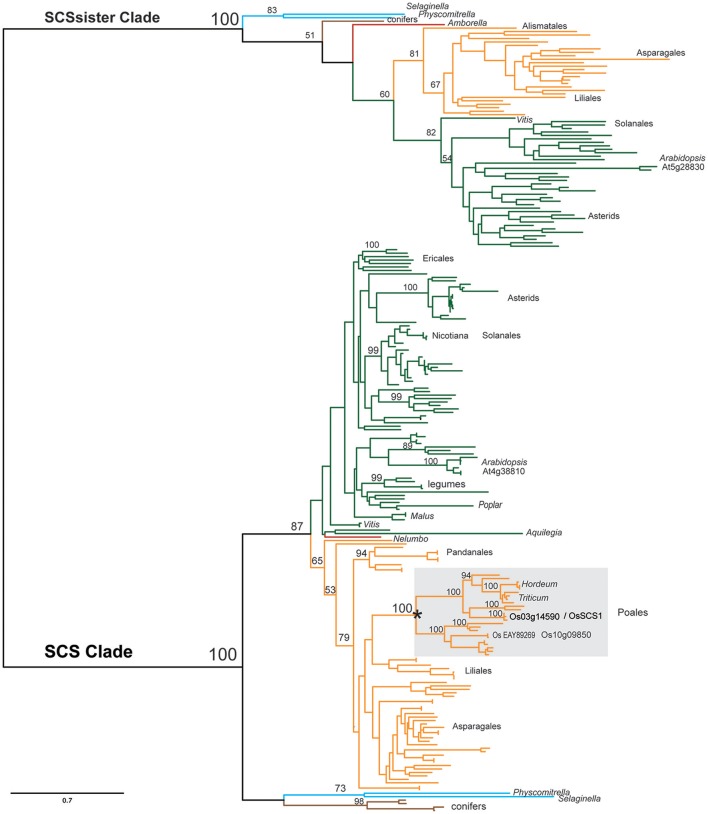
**Simplified SCS phylogeny**. Maximum likelihood (ML) phylogeny of SCS and sister clades inferred from 231 taxa and 660 amino acid characters. The detailed tree is presented in Supplementary Figures 3A–C. ML bootstrap values above 50% are shown above key clade branches. Orange, green, red, and teal colored branches denote monocot, dicot, *Amborella/Nelembo*, and *Selaginella moellendorfi/Physcomitrella patens* sequences, respectively. Poales representatives (gray rectangle) are not found in sister clade. Asterisk denotes polyploid duplication event. First letter of Genus and species and protein accession numbers are provided for each sequence. The list of taxa and their multiple sequence alignment used in this phylogeny are provided in Supplementary Table 5 and Supplementary File 4 in Phylip data format.

To more thoroughly explore the evolution of the SCS clade relative to its sister clade during plant evolution, we assembled an additional matrix, dataset4, using sequences from genomic (Kersey et al., 2016) and the 1KP transcript databases (Matasci et al., 2014). Figure 4 shows a summarized version of the detailed phylogenetic tree found in Supplementary Figures 3A–C. Each clade, defined by the Arabidopsis homolog of SCS (At4g38810) or SCSsister (At5g28830), is composed of a single major lineage that was established before the origin of land plants, as each contain homologs from mosses, lycopods, gymnosperms, and angiosperms. Within the SCS clade, there are relatively few ancient duplications, although at least one event (marked by an asterisk) appears to have occurred in the common ancestor of grasses, possibly in conjunction with the genome duplication known to be associated with this radiation (Yu et al., 2005). This event gave rise to the two rice loci: *OsSCS1* (Os03g14590) and its paralog Os10g09850 (65% identity). Evidence of very recent duplication events (marked by an asterisk) is also seen in known polyploids, such as *Gossypium* and *Triticum*. The SCSsister clade is separated from the SCS lineage with high support. Interestingly, no homolog of the sister clade was identified in the Poales, although they were detected in other monocot lineages.

### Putative rice TtPK1/OsSAPK2 calcium sensor partner: OsSCS1

**Figure 5:**
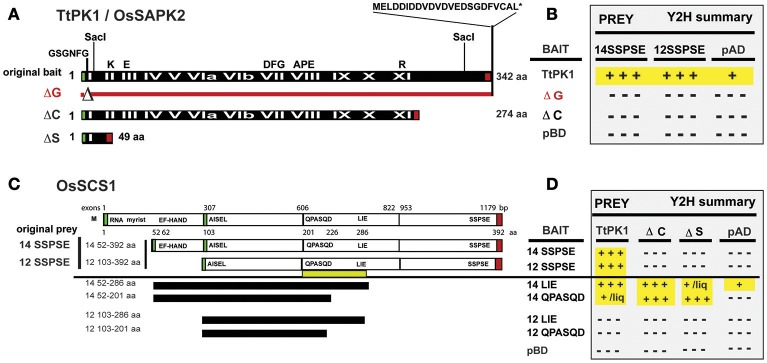
**Y2H screen for TtPK1/OsSAPK2-interacting protein: OsSCS1. (A)** Schematic of TtPK1/OsSAPK2. The “original bait” was full-length TtPK1 coding region (black bar) and has 100% amino acid identity with OsSAPK2. Roman numerals (in white text) identify 12 kinase catalytic subdomains. Black letters (above black bar) show relative locations of conserved kinase domain residues and the aspartic acid-rich C-terminus. Red line with triangle (ΔG) depicts the relative location of the glycine-rich nucleotide-binding amino acids (GSGNFG) that were removed to construct a catalytically dead kinase. The C-terminal mutant (ΔC) is truncated beyond kinase subdomain IX. The kinase domain mutant (ΔS) lacks the coding region between two SacI sites. Green and red boxes show locations of translational start and stop sites, respectively, for each peptide. **(B)** Summary of confirmed Y2H interactions. N-terminal BAIT BD fused to TtPK1, ΔG, or ΔC (left column) were in screening vector (pXDGATcy86) or in pGBKT7. N-terminal PREY AD fused to 14SSPSE or 12SSPSE were in screening vector, pAD-GAL4-2.1. Empty vector controls are pBD and pAD. Positive signs (highlighted in yellow) indicate “strong” dimerization, as inferred by abundant (+++) or little (+) growth. Negative signs indicate no interaction, as inferred by no growth. Results are detailed in Supplementary Figure 4 and Section Materials and Methods. **(C)** Schematic of OsSCS1. The Os03g14590 locus with five exons (white rectangles), interspersed with one long (1,778 bp), and three shorter introns (not shown) encodes predicted polypeptide OsSCS1 (top bar). Relative nucleotide positions of exon/intron junctions and amino acid positions are indicated above and below OsSCS1, respectively. Green and red boxes identify relative translation start and stop sites, respectively. The “original prey” cDNA clones (14 and 12) with complete 3′ ends are designated 14SSPSE or 12SSPSE, respectively, for their final C-terminal residues. Deletion Y2H constructs (black horizontal bars) were derived from “original prey” (14 or 12) and designated by their C-terminal residues: LIE or QPASQD. Relative positions of N- and C-terminal residues for each truncated version of OsSCS1 are indicated (left column). Yellow bar shows region between QPASQD and LIE required for dimerization. **(D)** Summary table of interactions. N-terminal BAIT BD fused to versions of OsSCS1 (left column) were in pGBKT7. N-terminal PREYAD-fused to TtPK1, ΔC, or ΔS (column headers Figure 5A) were in pGADT7. Empty vector controls are labeled pBD and pAD. Positive signs (highlighted in yellow) designate “strong” dimerization, as inferred by abundant (+++) or little (+/liq) growth. Negative signs indicate no interaction, as inferred by no growth. Some growth was observed in liquid (liq) culture after 16 h. Results in this table are detailed in Supplementary Figure 5 and Section Materials and Methods.

The TtPK1, which has 100% amino acid identity with its rice ortholog, OsSAPK2, was used as “original bait” (Figure 5A) to screen yeast two-hybrid (Y2H) libraries prepared from ~14 to 21 day old rice seedlings (*Oryza sativa indica* cv IRBB21) that were treated with abiotic and biotic stresses (Seo et al., 2011). Our Y2H screen recovered two TtPK1-interacting proteins: as expected, the rice ortholog (OsABI5) and the *Oryza sativa SnRK2*-interacting calcium sensor (*OsSCS1*). We recovered 12 independent yeast Y2H clones that appeared to encode OsSCS1; predicted polypeptides of only two partial clones had an N-terminal EF-hand motif. Figure 5C shows a schematic of the original prey cDNA deduced peptides: 14 and 12.

By comparing their sequences to the predicted genomic *OsSCS1* locus (Os03g14590) from rice (*Oryza sativa* indica and japonica) databases, we determined that two original prey cDNAs (14 and 12) were incomplete at their 5′ ends. Relative to the inferred start methionine, clone 14 lacks 51 amino acids while clone 12 lacks a total of 102 amino acids. The predicted peptide of clone 14 had a conserved N-terminal calcium-binding EF-hand motif (DADGDGRLSVSEL), which was missing from clone 12. The indicated methionine is likely to be the correct start methionine because just 12 nucleotides upstream is an in-frame stop codon. Clones 14 and 12 do appear to be complete at their 3′ ends due to the presence of poly-adenylated tails. For this study, we have termed the original prey predicted proteins 14SSPSE and 12SSPSE, for the final five residues of their coding regions (Figure 5C).

### TtPK1 and OsSCS1 C-termini are responsible for dimerization

We first examined the regions of the kinase and OsSCS1 necessary for the protein-protein interactions. In Figures 5A,B, we show a summary of our Y2H experiments that are detailed in Supplementary Figure 4. The Y2H screening vectors were used initially to demonstrate the “strong” affinity of the two full-length kinases (TtPK1 and PKABA1) with the “original prey” peptides (14SSPSE and 12SSPSE), as inferred by the abundant growth even with the addition of high levels histidine competitive inhibitor 3-AT. Growth was minimized with addition of 20 and 30 mM 3-AT. However, the catalytically dead kinase construct TtPK1 (ΔG), which lacked the conserved nucleotide binding site GSGNFG, did not interact with either 14SSPSE, or 12SSPSE. No growth occurred even with the least restrictive media (−ALT or −HLT with no 3-AT). Liquid growth and α-galactosidase secretion assays confirmed the strong Y2H protein interactions, with OsSCS1/kinase increased 7.5- to 30-fold from non-interaction controls.

We knew that TtPK1/OsSAPK2 has a conserved glycine-rich nucleotide-binding site (GSGNFG), as well as a stretch of 10 acidic amino acid residues beyond the catalytic subdomain (Figure 5A, Supplementary Figure 4A). We hypothesized that both were necessary for the interaction with the C-terminal region of OsSCS1. This was based on our previous studies with catalytically dead kinase constructs (ΔG) of PKABA1 and TtPK1, which indicated that the conserved nucleotide binding site was essential for their transient functional activity (Gómez-Cadenas et al., 1999; Holappa et al., 2005) and for their interaction with TaABF (Johnson et al., 2002). As the majority of the recovered Y2H prey clones were those of 12SSPSE, we also reasoned that the C-terminal region of the OsSCS1 polypeptide was necessary for the interaction, but the predicted N-terminal EF-hand was not absolutely required.

To test whether the C-terminal end of TtPK1 is required for the interaction, we prepared a truncated TtPK1 construct by removing the final 68 residues (abbreviated ΔC). As expected, we observed “strong” growth when full-length TtPK1 was paired with either 14SSPSE or 12SSPSE (Figures 5B,D, Supplementary Figures 4B–D). However, when TtPK1 lacked its C-terminal end (ΔC), we observed no growth with 14SSPSE, which contained the N-terminal EF-hand, and some growth with 12SSPSE. Supplementary Figure 4D shows representative protein interactions of a complete Y2H experiment that was assayed for growth after 4 days on plates containing non-selective (-LT) and selective (-HLT with 10 mM 3-AT) dropout SD media. Homo-dimerization of TtPK1 was not detected for any of the combinations (Supplementary Figure 4E). Some growth by autoactivation occurred with the GAL4-BD-TtPK1 fusion protein but was minimized by the addition of 3-AT and the removal of adenine and histidine from the media (−AHLT). A complete dilution series on restrictive media, as in Supplementary Figure 4C, was conducted for assays in Supplementary Figures 4D,E (data not shown).

We next examined the regions of OsSCS1 that are necessary for the protein-protein interaction. Figures 5C,D shows a schematic and summary of our detailed Y2H analyses in Supplementary Figure 5. To test whether the OsSCS1 C-terminal region was required for the kinase interactions, we prepared several deletion constructs by PCR-modifying the “original prey” peptides (14SSPSE or 12SSPSE) recovered from the Y2H screen. Specifically, we removed the C-terminal regions beyond the residues LIE, designated 14LIE (52–286) and 12LIE (103–286), and also beyond the residues QPASQD, designated 14QPASQD (52–201) and 12QPASQD (103–201) (Figure 5C). We did not test the N-terminal residues missing from 14SSPSE as they were not present in the original Y2H clones and are, by default, not essential to the interaction. As positive controls, we included the original screening protein pairs in this comprehensive Y2H assay (Supplementary Figure 5C bottom panel). All protein interactions were inferred by growth of host AH109 yeast after 4 days on non-selective (−LT) and selective (−HLT, −ALT, −AHLT) dropout SD media.

We found that growth (i.e., dimerization) always occurred when the N-terminal EF hand was present (14LIE, 14QPASQD) even without the C-terminal end (ΔC) or the kinase subdomains (ΔS). When OsSCS1 was C-terminally truncated by 106 amino acids (14LIE) abundant growth still occurred, inferring “strong” interaction with the full-length kinase. However, when OsSCS1 was severely C-terminally truncated by 191 amino acids (14QPASQD), no growth occurred, thus inferring no interaction with full-length kinase. These findings show that the region between LIE and QPASQD (underscored by yellow bar in Figure 5C, and motif 5; Figure 3) is absolutely necessary for OsSCS1 dimerization, as long as the N-terminal EF-hand is present. Without the N-terminal EF-hand, 12LIE or 12QPASQD, no interactions occurred as inferred by no growth (Figures 5C,D). Our liquid Y2H assays, which measured the secretion of β-galactosidase activities into liquid selective SD media following 18-h of yeast growth, confirmed the results observed with plated yeast (Supplementary Figure 5C, right panel).

### Co-immunoprecipitation and split-YFP confirm the TtPK1/OsSCS1 interaction

To confirm the Y2H interaction, we produced constructs that contained T7-tagged versions of OsSCS1 or non-tagged, ^35^S-met-labeled-TtPK1 for co-immunprecipitation (coIP) assays, following (Wang et al., 2002). We summarize our results in Supplementary Figure 6. Briefly, we expressed *in vitro* each recombinant protein separately in a rabbit reticulocyte (RRL) transcription and translation system and then stopped each reaction with cyclohexamide. Each recombinant protein was expressed at approximately the same level and was soluble, as shown in a representative Coumassie-stained gel (Supplementary Figure 6C). We then independently tested the ability radioactively labeled TtPK1 to interact with each T7-tagged OsSCS1 peptide (14SSPSE, 12SSPSE, 12QPASQD, 12Δ) by co-IP with T7-tag antibody coupled to Protein A Sepharose. Supplementary Figures 6E–G shows that precipitated non-tagged ^35^S-met-TtPK1 corresponds to a band of ~38 kD, after size-fractionation by PAGE and image analysis from the phosphor-screen (Supplementary Figures 6E–G). Non-tagged ^35^S-met-TtPK1 did not precipitate with beads (^**^) or empty vector (pET, pC). When the binding partners were reversed, T7-TtPK1-bound-antibody precipitated with the non-tagged ^35^S-met-Ca14SSPSE (14), corresponding to a band of ~38 kD after size-fractionation by PAGE and image analysis of the phosphor-screen (Supplementary Figure 6F, lane 8). In a replicate experiment with independent reactions, T7-Ca14SSPSE-bound-antibody precipitated with the non-tagged ^35^S-met-TtPK1 (lanes 3 and 4) and co-migrated with input ^35^S-methinone-labeled TtPK1 (lane 10), after size-fractionation with PAGE and imaging of phosphor-screen.

**Figure 6:**
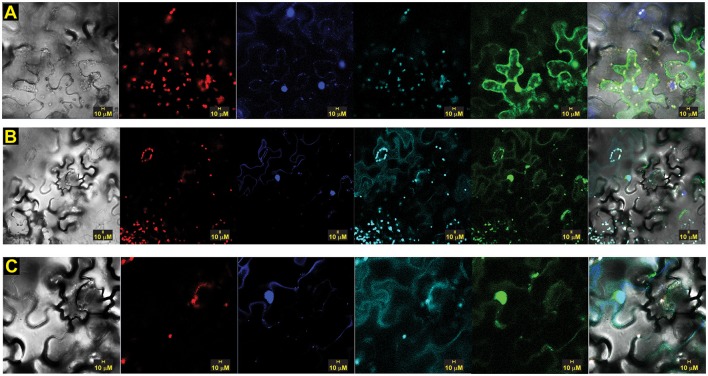
**Split-YFP dimerization of OsSCS1-OsSAPK2/TtPK1 is nuclear and cytosolic**. Subcellular localization of bimolecular fluorescence complementation (BiFC) of OsSCS1 and TtPK1 in *N. benthamiana* leaves transiently expressed from co-infiltrated *Agrobacterium*-transformed constructs. Representative images show the dimerization of: **(A)** N-terminal YFP-OsSCS1 and C-terminal YFP-TtPK1, **(B)** a second independent construct of N-terminal YFP-OsSCS1, and C-terminal YFP-TtPK1 at higher magnification **(C)**. Columns from left to right are images captured by bright-field, chloroplast auto-fluorescence, DAPI-stain, FM4-64 stain, GFP, and overlay. At least three independent infiltration experiments were conducted for each construct with 10–15 leaf sections observed and 5–10 images recorded.

To confirm the interaction and assess its intracellular location *in planta*, we conducted transient bimolecular fluorescence complementation (BiFC) experiments using constructs containing the N-terminal domain of yellow fluorescent protein (YFP) fused to OsSCS1 and the C-terminal domain of YFP fused to TtPK1. For the OsSCS1 construct, we first completed its likely coding region by adding 153 nucleotides to the 5′end of the 14SSPSE cDNA using successive rounds of PCR. When co-expressed in abaxial epidermal layers of *N. benthamiana* leaves 2–3 days after *A. tumefaciens*–mediated leaf transient transformation, we observed fluorescence from the complemented split YFP domains, indicative of the interaction between OsSCS1 and TtPK1 (Figure 6). Interestingly, the YFP signal accumulates in the nucleus, as confirmed by its co-localization with DAPI nucleic acid stain, and also around cytosolic membranes stained with FM4-64. This lipophilic dye is used initially to monitor the plasma membrane before internalized by endocytic trafficking (Vida and Emr, 1995; Bolte et al., 2004; Wang et al., 2010). We believe that YPF signal is likely to be the result of the OsSCS1/TtPK1 protein partner interaction and not just the re-assembly of the split YFP domains because our parallel experiments with empty vectors yielded diffuse or non-detectable YFP signals (Supplementary Figure 7).

### OsSCS1 has nuclear and cytosolic distributions and when co-expressed with TtPK1

**Figure 7:**
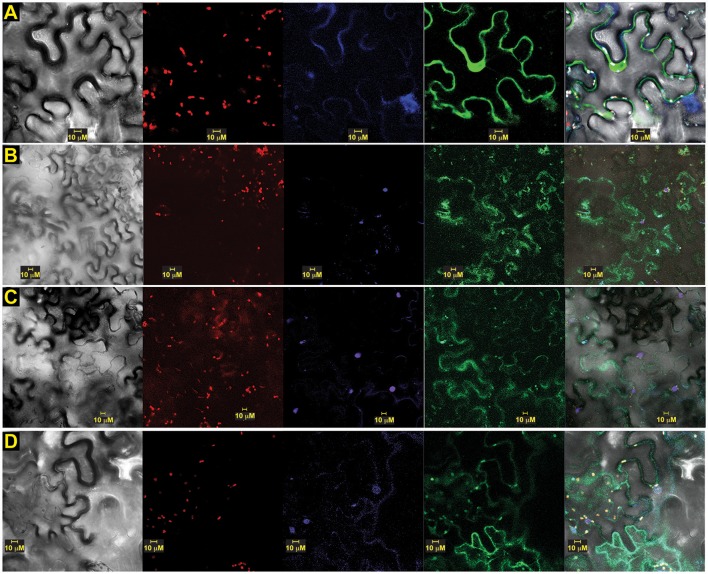
**OsSCS1 distribution is nuclear and cytosolic around chloroplasts**. Subcellular localization of versions of OsSCS1 in *N. benthamiana* leaves transiently expressed from a singly infiltrated *Agrobacterium*-transformed construct. Representative images show the expression of: **(A)** N-terminal GFP-OsSCS1, **(B)** N-terminal GFP-14SSPSE, **(C)** 14SSPSE C-terminal GFP, and **(D)** empty-vector GFP control. Columns from left to right are images captured by bright-field, chloroplast autofluorescence, DAPI-stain, GFP, and overlay. At least three independent infiltration experiments were conducted for each construct with 10–15 leaf sections observed and 5–10 images recorded. In **(A)** the DAPI-stained nucleus in lower right corner overlaps with OsSCS1-GFP.

To assess the calcium sensor's intracellular location, we also prepared translational fusion constructs with green (G)FP-tagged to versions of OsSCS1, all driven by cauliflower mosaic virus (CaMV) 35S promoter. When expressed in abaxial epidermal layers of *N. benthamiana* leaves 2–3 days after *A. tumefaciens*-mediated leaf transient transformation, green (G)FP-tagged versions of OsSCS1 were expressed in the nucleus and cytoplasm (Figure 7). To assess localization, we also infiltrated the leaves with the nucleic acid stain DAPI just prior to imaging. Figure 7A shows a representative image of N-terminal GFP-OsSCS1 is distributed in the cytosol surrounding the large central vacuole and also in a DAPI-stained nucleus (Figure 7A lower right hand corner). Similarly, N-terminal GFP-14SSPSE (original prey) is nuclear but mostly cytosolic (Figure 7B). To account for the possibility of only N-terminal GFP being expressed due to a truncated translation, we tested constructs containing 14SSPSE fused C-terminally to a GFP-tag. This peptide fusion rarely co-localized to the DAPI-stained nuclei (Figure 7C). When we tested constructs containing of N-terminal GFP-12SSPSE (original prey), which lacked the N-terminal EF-hand, the peptide fusion accumulated only in the cytosol, appearing as membrane projection around chloroplasts (Supplementary Figures 8A,B). For a nuclear control, we also transiently expressed constructs containing N-terminal GFP fused to three nuclear localization signals (NLS) followed by DAPI staining (Supplementary Figures 8C,D).

**Figure 8:**
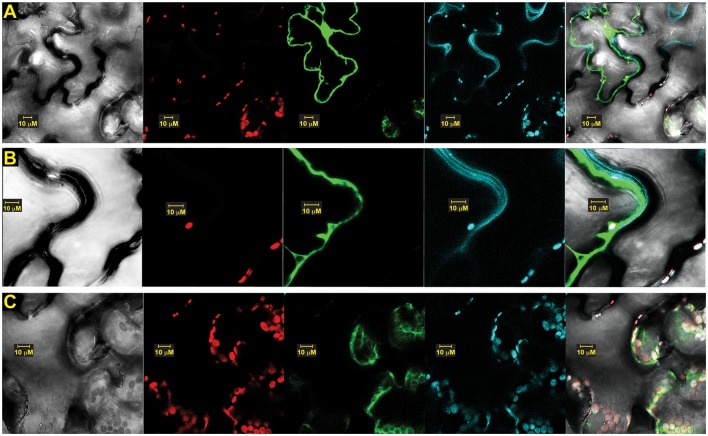
**OsSCS1 cytosolic distribution is around plasma membrane and chloroplasts**. Subcellular localization of OsSCS1 in *N. benthamiana* leaves transiently expressed from a singly infiltrated *Agrobacterium*-transformed construct. Representative images show expression of: **(A)** N-terminal GFP-OsSCS1 at higher magnification along plasma membrane **(B)** and chloroplasts **(C)**. Columns from left to right are images captured by bright-field, chloroplast auto-fluorescence, FM4-64 stain, GFP, and overlay. At least three independent infiltration experiments were conducted for each construct with 10–15 leaf sections observed and 5–10 images recorded.

To determine whether versions of OsSCS1 associated with the plasma membrane, we also infiltrated the leaf section with the styryl dye FM4-64 just prior to imaging. Figure 8A shows a representative image of N-terminal GFP-OsSCS1 expressed in the nucleus and cytosol around the large vacuole. The cell has also been stained with the lipophilic dye FM4-64, which co-localizes along the resolved membranes (Figure 8B) and around chloroplasts (Figure 8C).

**Figure 9:**
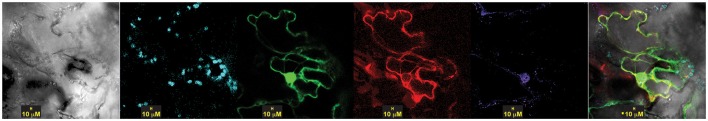
**TtPK1 and OsSCS1 co-distribution is nuclear and cytosolic, around chloroplasts**. Subcellular of localization in *N. benthamiana* leaves transiently expressed from co-infiltrated *Agrobacterium*-transformed constructs: N-terminal GFP-TtPK1 and OsSCS1-C-terminal RFP Columns from left to right are images captured by bright-field, chloroplast auto-fluorescence, GFP, RFP, DAPI-stain, and overlay. At least three independent infiltration experiments were conducted for each construct with 10–15 leaf sections observed and 5–10 images recorded.

To assess the intracellular location of OsSCS1 with the kinase TtPK1, we prepared OsSCS1 fused C-terminally to a red (R)FP-tag and co-expressed it with an N-terminal GFP-tag fused to the kinase, TtPK1. Figure 9 shows a representative image of the GFP-kinase peptide expressed in the nuclei and cytosol, consistent with our previous report of TtPK1 in aleurone protoplasts (Holappa et al., 2005) and with reports of other SnRK2 orthologs (Mizoguchi et al., 2010; Tian et al., 2013). The same cell with OsSCS1-RFP co-expressed in cytosol and around the nucleus co-stained with DAPI.

### Dehydration increases the levels of *OsSCS1*-hybridizing transcripts

To ascertain whether *OsSCS1* is responsive to dehydration stress, we examined *OsSCS1*-hybridizing transcripts in 14 days-old rice (*O. sativa indica*) seedlings that were germinated and grown in fully hydrated conditions and then allowed to dry for 16 h in a petri dish without water. Supplementary Figure 9A shows a schematic of the *Os03g14590* locus with its predicted mRNA and region used for the ^32^P-labeleled cDNA probe (underlined in red). Supplementary Figure 9B shows elevated levels of *OsSCS1*-hybridizing transcripts of ~1.4 kb in shoot/coleoptiles of drying seedlings (DRY) relative to those kept fully hydrated (H_2_O), after a 3- and 60-h exposure of the blot to the phosphor screen. At 60 h, a larger ~3.5 kb transcript was also present, likely corresponding to a partially processed transcript that still contains the large first intron. Note that we utilized RNAblot technology in these preliminary expression analyses to assess alternatively processed longer transcripts, which are not usually amplified using traditional PCR methods.

When the seedling experiment was repeated to examine earlier time points post-dehydration and *OsSCS1-*relative to *TtPK1*-hybridizing transcripts (Supplementary Figure 9C), *OsSCS1*-hybridizing transcripts were already present in crown tissues at 0, 2, 4, and 8 h of dehydration, although the ~3.5 kb transcript was markedly decreased after 8 h of treatment. When we examined the shoot/coleoptile tissues, we observed a large accumulation of the ~1.4 kb OsSCS1 transcript at 2 h and subsequent time points. The ~3.5 kb transcript was present at low levels at all-time points (Supplementary Figure 9C). Hybridization of *TtPK1* probe to the same blot after 60-h exposure revealed that the kinase-hybridizing transcripts co-accumulated with *OsSCS1*-hybridizing transcripts within 2 h post-dehydration and both were maintained at elevated levels at subsequent time points (Supplementary Figure 9C). Larger ~3.5 kb *TtPK1*-hybridizing transcripts were also present, likely corresponding to partially processed kinase transcripts that still contain larger first and second introns.

## Discussion

Early phylogenetic studies of Arabidopsis kinases recognized similarities between the calcium-dependent protein kinases and the SNF1-related kinases (collectively termed the CDPK-SnRKs) based on comparisons of only catalytic domains, and suggested that these kinases are regulated by environmental responses mediated by calcium (Hrabak et al., 2003). Later, a series of analyses divided the Arabidopsis and rice SnRK2 kinases into three groups based on sequence similarities and on their activation (i.e., their *in gel* phosphorylation of peptides) in response to ABA and/or osmotic stress treatments (Kobayashi et al., 2004; Boudsocq and Lauriére, 2005; Umezawa et al., 2010; Kulik et al., 2011). Although nomenclature for the groups has varied among publications, kinases in what are termed Subclass I are activated only by osmotic stress and not by ABA (e.g., OsSAPK4-7, AtSnRK2.1/4/5/9/10); Subclass II are activated weakly by ABA and by osmotic stress (e.g., OsSAPK1-3, AtSnRK 2.7, 2.8); and Subclass III are strongly activated by ABA and osmotic stress (e.g., OsSAPK8-10, AtSnRK 2.2, 2.3, 2.6). Arabidopsis with single and multiple SnRK2 mutations have confirmed the dissected responses among subclasses and their redundancy in osmotic stress responses, whether or not ABA-dependent (Mizoguchi et al., 2010; Fujii et al., 2011; Fujii and Zhu, 2012).

Recent studies with maize representatives have suggested the possibility of four SnRK2 clades (Vilela et al., 2012). Our SnRK2 phylogenetic analysis using Maximum Likelihood (Figure 1, Supplementary Figures 1, 2) is the most broadly sampled and rigorous to date. It clearly demonstrates the presence of four pan-angiosperm SnRK2 clades, each of which possesses at least one core eudicot (e.g., *Populus*), one early diverging angiosperm (e.g., *Amborella*), and one monocot (e.g., *Oryza*) representative. We have simply named the four clades based on their rice representatives. Relative to the nomenclature above, there is a one to one correspondence between Subclass I and our OsSAPK4-7 clade, and Subclass III and our OsSAPK8-10, but Subclass II encompasses our distinct clades OsSAPK1/2 and OsSAPK3.

The two distinct OsSAPK1/2 and OsSAPK3 clades share similar responses to ABA and osmotic stresses, even though the *OsSAPK3* coding region has a considerably shorter C-terminus. In terms of the SAPK3 clade, Os*SAPK3* mRNA, originally called rice endosperm kinase (REK), was cloned from 15-d post-flowering grains, was shown to be present in seed and leaf tissue, and its recombinant protein required Ca^2+^ to auto-phosphorylate (Hotta et al., 1998). Although OsSAPK3 and OsSAPK1/2 transcripts accumulate in response to ABA and osmotic treatments, the other seven *OsSAPK* mRNAs do not (Kobayashi et al., 2004). Also, in rice culture cells, OsSAPK3 and OsSAPK1/2 are activated (i.e., phosphorylated) weakly in response to ABA and strongly by osmotic treatments. The significance of the OsSAPK3 clade remains unclear but its distinct pattern of sequence evolution suggests that loss-of-function and/or overexpression studies are needed to explore their function.

Although it has been suggested that the SAPK8/9 clade is the “oldest” subfamily (Mizoguchi et al., 2010), this is not supported by the data. While the *Physcomitrella* and *Selaginella* homologs are associated with this clade, these relationships would actually suggest that at least two lineages were established before the diversification of land plants: the SAPK8/9 lineage and one or more ancestral lineages that gave rise to the other clades. During land plant diversification, it would appear that homologs of OsSAPK8/9 were retained in the *Selaginella* and *Physcomitrella* genomes while any other representatives were lost. However, we recognize that support for the position of the non-seed plant homologs with OsSAPK8/9 is marginal. This raises other possible evolutionary scenarios, such as a model where the non-seed plant sequences are sister to all four seed plant subfamilies. The rapidly growing availability of non-angiosperm transcriptome datasets will provide the much needed sampling that would be necessary to resolve this question.

As for SCS evolution, our unrooted phylogenetic tree shows that the SCS and SCSsister clades are distinct from the shorter related clades, SRCs (Figure 2) even though they share conserved features, such as putative signature EF-hand motifs and stretches of multiple prolines (Figure 3). With additional monocot and basal eudicot (e.g., *Amborella, Nelumbo*) sequences from transcriptomes, our detailed analyses have improved the resolution relative to the sister clade (Figure 4, Supplementary Figures 3A–C). All members of the SCS lineage have conserved features that include putative signature EF-hand motifs, stretches of multiple prolines, a putative N-terminal myristoylation motif, and a possible basic RNA binding domain. Not surprisingly, both rice SCS paralogs (Os03g14590 and Os10g09850) could interact with seven OsSAPKs, suggesting these SCSs have a similar or redundant cellular function (Ding et al., 2009).

Although the clade of SCSsister sequences is distinctly segregated from the SCS lineage with high support (Figure 4, Supplementary Figures 3A–C), it lacks homologs from the Poales while it contains sequences from other monocots and core eudicot representatives. The significance of the SCSsister clade remains unclear but the fact that it has conserved C-terminal sequences with the main OsSCS1 lineage (Figure 3) raises questions as to whether these proteins may also be capable of interacting with SnRK2 partners. The broad angiosperm, conservation of SCS/SCSsister C-terminal motifs shown here to be important for SnRK2 interactions suggests that these EF-hand containing proteins may have a common role in the regulation of these critical stress response kinases.

### OsSCS1: interacting protein of TtPK1/OsSAPK2

We sought protein interacting partners in seedling leaves for TtPK1, and by extension for its identical protein ortholog, OsSAPK2. As such, we obtained a putative SnRK2 calcium sensor protein from *Oryza sativa*, termed OsSCS1, which also interacts with seven OsSAPK homologs of the SnRK2 subfamily (Ding et al., 2009) and is orthologous to the previously identified eudicot SnRK-interacting calcium sensor (SCS) proteins (Bucholc et al., 2011). Our RNA blot analyses showed that *OsSCS1*, like *TtPK1* and *PKABA1*, is transcriptionally regulated by dehydration (Supplementary Figure 9), consistent with similar stimuli that regulate SnRK2 homolog expression and activity (Kobayashi et al., 2004; Holappa et al., 2005). Our yeast two hybrid (Y2H) analyses (Figure 5, Supplementary Figures 4, 5) confirmed that the OsSCS1 interaction with TtPK1/OsSAPK2 requires the six residues of N-terminal nucleotide binding. This was expected as our previous work with PKABA1 and TtPK1 showed that the nucleotide-binding motif is required for transcriptionally suppressing gibberellic acid (GA)-inducible α-amylase promoter in barley aleurone and is also necessary for the interaction between PKABA1 and *Triticum aestivum* ABA binding factor, TaBF (Gómez-Cadenas et al., 1999; Johnson et al., 2002; Holappa et al., 2005).

Our detailed Y2H experiments also revealed that the removal of the OsSCS1 C-terminal region along with its N-terminal EF-hand abolished the interaction with TtPK1/OsSAPK2 and that a middle stretch of OsSCS1 is necessary for interaction (Figure 5). The finding that TtPK1 and other OsSnRK2 homologs may interact *in planta* with OsSCS1 via their critical C-terminal domains suggests a role in modulating SnRK2 activities (Kobayashi et al., 2004; Kulik et al., 2011). As previous studies with rice SAPKs have demonstrated (Kobayashi et al., 2004), removal of the stretch of C-terminal acidic residues reduces the activities of Subclass II kinases in cereal clades SAPK1/SAPK2 and SAPK3. Ongoing research has established that the C-terminal domains of Subclass III SnRK2s (2.2, 2.3, 2.6) are critical for their ABA-mediated activation by PP2C phosphatases (Kobayashi et al., 2004; Nishimura et al., 2010; Soon et al., 2012). Similarly, a subclass III maize ortholog (ZmSnRK2.8) is also activated by ABA and its C-terminal regulatory region interacts with maize PP2C phosphatase (Vilela et al., 2012). More recently, maize casein kinase 2 (CK2) phosphorylated the C-terminal ABA box of maize subclass III ortholog ZmSnRK2.8 (renamed ZmOST1) increasing PP2C binding (Vilela et al., 2015).

We also show that the dimerization of TtPK1/OsSAPK2 and OsSCS1 occurs in both the nucleus and cytosol, while versions of OsSCS1 alone are mostly cytosolic, implicating these sites for their functions. We hypothesize that the occasional nuclear localization may be anomalous due to only the fluorescent protein (Schornack et al., 2009) or that some other controlled cellular process determines the intracellular location. For example, the Arabidopsis ortholog of OsSAPK2 phosphorylates protein partners, NTL6 and NPR1, contributing to their nuclear import and to their responses in drought-stress and pathogens, respectively (Kim M. J. et al., 2012; Lee et al., 2015).

Our study builds on a growing body of data demonstrating that SnRK2 homologs participate in multiple protein–protein and small molecule interactions that are likely to be critical to the integration of endogenous developmental signals (e.g., ABA, Ca^2+^ flux) with exogenous environmental signals (e.g., dehydration, cold, light) reviewed in Fujii and Zhu (2012), Mogami et al. (2015), and Yoshida et al. (2015). For example, quantitative phosphor-proteomic assays have identified many new SnRK2 substrates, including those involved in metabolism, flowering time regulation, nuclear-encoded chloroplast pre-proteins transport, and chromatin-remodeling (Shin et al., 2007; Wang et al., 2013).

The fact that the SnRK2/SCS interactions are conserved among monocots, grasses, and core eudicot model systems is consistent with the high conservation of *SCS* homologs across the angiosperms, and suggests that these EF-hand containing proteins may have a common role in the regulation of SnRK2s, particularly in stress responses (Edel and Kudla, 2015). Future studies are needed to determine how post-translational modifications and Ca^2+^ binding may interact to regulate SCS/SnRK2s and in turn a pleiotropic range of plant growth phases.

## Author contributions

Conceived and designed experiments: LH, PR, and EK. Conducted Y2H screen: LH. Conducted all experiments: LH. Analyzed data: LH and EK. Wrote paper: LH and EK. Revised drafts: PR. Contributed funding for reagents, materials, analysis tools: PR and EK.

### Conflict of interest statement

The authors declare that the research was conducted in the absence of any commercial or financial relationships that could be construed as a potential conflict of interest.
