# Supplementary material for: Evolutionary Analysis of Snf1-Related Protein Kinase2 (SnRK2) and Calcium Sensor (SCS) Gene Lineages, and Dimerization of Rice Homologs, Suggest Deep Biochemical Conservation across Angiosperms
Source: Front Plant Sci. 2017 Apr 5;8:395. doi: 10.3389/fpls.2017.00395 (PMC5381359; doi:10.3389/fpls.2017.00395)
Supplement: Supplementary file 2 [file SupplementaryFigureLegends.pdf]

Supplementary Material

Evolutionary Analysis of Snf1-Related Protein Kinase2 (SnRK2) and Calcium Sensor (SCS) Gene Lineages, and Dimerization of Rice Homologs, Suggest Deep Biochemical Conservation across Angiosperms

Lynn D. Holappa[*], Pamela C. Ronald, Elena M. Kramer

*** Correspondence:** Lynn D. Holappa: lholappa@oeb.harvard.edu

1 Supplementary Figures and Tables

1.1 Supplementary Figures

Supplementary Figure 1. SNF1-Related Kinase (SnRK) Superfamily Phylogeny. Maximum likelihood (ML) phylogeny of the yeast SNF1-related superfamily of kinases inferred from 246 taxa and 399 characters. Amino acid residues in N- and C- terminal regions that could not be confidently aligned were excluded from the multiple sequence alignment (MSA). ML bootstrap values above 70% are shown above key branches. The superfamily is rooted with the SnRK1 clade, which includes all fungal and animal SNF1 homologs. Orange, green, and teal colored branches denote representatives from monocots, dicots, and *Selaginella moellendorfi* and *Physcomitrella patens*, respectively. Grey rectangles identify three SnRK2 clades. Rice and Arabidopsis sequences are abbreviated with first letter of Genus and species and protein accession numbers. The list of taxa and their MSA used in this phylogeny are provided in Supplementary Table 2 and Supplementary File 1 in FASTA data format.

Supplementary Figures 2a-d. Detailed Phylogeny of Figure 1 shown in: (2a) OsSAPK3 and 1/2, (2b) OsSAPK8/9/10, (2c) OsSAPK4/5/6/7, (2d) OsSnRK1 and SNF1/AMPK. Maximum likelihood (ML) phylogeny of SnRK2 and SnRK1 related kinases inferred from 285 taxa and 335 amino acid characters, rooted relative to the SnRK1/SNF1/AMPK clade. The PASTA algorithm was used to obtain multiple sequence alignments (MSA) of complete polypeptide sequences. A simplified tree is presented in **Figure 1**. ML bootstrap values above 50% are shown above key clade branches. Four distinct SnRK2 clades are identified by grey rectangles. The first letter of Genus and species are identified in bold text for proteins specifically discussed in the text. Accession number, first letter of Genus, three letters of species, and amino acid length are provided for each sequence. Asterisks denote ploidy duplication events. Orange, green, red, and teal colored branches denote monocot, dicot, Amborella, and *Selaginella moellendorfi* and *Physcomitrella patens* representatives, respectively. The list of taxa and their MSA used in this phylogeny are provided in Supplementary Table 3 and Supplementary File 2 in FASTA data format.

Supplementary Figure 3a-c. Detailed Phylogeny of Figure 4 shown in: (3a) SCSsister, (3b) Dicot SCS, (3c) Monocot SCS. (Inset) Colored branches show location of clade(s) within the full phylogeny (**Figure 4**). Maximum likelihood (ML) phylogeny of SCS and related clades inferred from 231 taxa and 660 amino acid characters, rooted relative to the SnRK1/SNF1/AMPK clade.

Complete polypeptide sequences were aligned with the PASTA algorithm. ML bootstrap values above 50% are shown above branches. Accession number, first letter of Genus, three letters of species, and amino acid length are provided for each sequence. First letter of Genus and species are identified in bold text for proteins specifically discussed in the text. Asterisks denote ploidy duplication events. Orange, green, red, and teal colored branches denote monocot, dicot, Amborella/Nelumbo, and *Selaginella moellendorfi* and *Physcomitrella patens* representatives, respectively. The list of taxa and their multiple sequence alignment used in this phylogeny are provided in Supplementary Table 5 and Supplementary File 4 in FASTA data format.

Supplementary Figure 4. Yeast Two Hybrid Screen for TtPK1-Interacting Protein: OsSCS1 (A) Schematic of TtPK1/OsSAPK2. **(B)** Y2H Summary. See text and Figure 5. **(C) Confirmation Y2H assay.** N-terminal GAL4BD fusions with TtPK1, ΔG, or PKABA1 in screening "bait" vector (BD column) were tested for interaction with N-terminal GAL4AD fusions to indicated OsSCS1 version in screening "prey" vector (AD column). See Figure 5A, C for peptide descriptions. All Y2H assays were conducted in triplicate, as described in manufacturer's protocols with modifications in Materials and Methods. After 18 h incubation (shaking at 30°C), each AH109 culture was diluted to OD600 of 0.5 and this same diluted culture was used for both the liquid and plating Y2H assays. **Left panel.** Protein interactions were assayed for growth on plates containing nonselective and restricted SD dropout media after 4 days. The LT column shows a representative colony of transformed AH109 strain plated on nonrestrictive synthetic dropout (SD) media (-LT), confirming viability. The HLT, ALT, and AHLT columns show dilution series (10^{0}, 10^{-1}, and 10^{-2}) of each strain grown on SD media, lacking histidine (H), leucine (L), tryptophan (T), and adenine (A), and supplemented with 0, 10, 20, or 30 mM 3-amino-1, 2, 4-triazole (3-AT), as indicated by the number before HLT or AHLT. Manufacturer's controls: positive (p53/T-antigen) and negative (empty/CL1, LAM/empty); and a positive control of *Aquilegia* MADS proteins (AqAP31/ AqPI) were also included. Images are representative plates from one of the three replications of the plate assay. **Right panel.** Protein interactions were monitored by secreted α-galactosidase (α-GAL) activity in liquid SD media -HLT plus 20 mM 3-AT media following 18 h of AH109 growth. Triplicate cultures and assays were conducted. Error bars are shown. **(D) Test for the necessity of the TtPK1-C-terminus.** N-terminal GAL4BD fusions with TtPK1 or ΔC in pGBKT7 (BD column) were tested for interaction with N-terminal GAL4AD fusions to indicated OsSCS1 version in screening prey vector (AD column). Columns of are colonies from dilution series (10^{0}, 10^{-1}, and 10^{-2}) of each strain on non-restrictive SD media (LT column) or selective SD media –HLT with 10 mM 3-AT (10HLT column). Shown are representative plates from the complete Y2H dilution series on both non-restrictive and selective media, which was conducted as in Supplementary Figure 4C. Labels are the same as panel C. **(E) Test for homo-dimerization.** N-terminal GAL4BD fusions with TtPK1 or ΔG in pGBKT7 (BD column) were tested for interaction with N-terminal GAL4AD fusions with TtPK1, ΔC, or ΔS in pGADT7 (AD column). Columns of are colonies from dilution series (10^{0}, 10^{-1}, and 10^{-2}) of each strain on non-restrictive SD media (LT column) or selective SD media –HLT with 10 mM 3-AT (10HLT column). Shown are representative plates from the complete Y2H dilution series on non-restrictive/selective media was conducted, which was conducted as in Supplementary Figure 4C. Labels are the same as in panel C.

Supplementary Figure 5. **C-terminal Ends of TtPK1 and OsSCS1 are Required for Dimerization. (A)** Schematic of OsSCS1, (**B)** Y2H summary. (**C) Y2H assay**. N-terminal GAL4BD fusions with indicated peptide in pGBKT7 (BD column). N-terminal GAL4AD fusions to indicated peptide in pGADT7 (AD column). Versions of OsSCS1 (designated: 14LIE, 14QPASQD,

12LIE, or 12QPASQD) and TtPK1 (designated: ΔC or ΔS) are as described in **Figure 5.** N-terminal GAL4AD fusions of 14SSPSE and 12SSPSE are in the screening “prey” vector. After 18 h incubation (shaking at 30°C), each AH109 culture was diluted to OD600 of 0.5 and this same diluted culture was used for both the liquid and plating Y2H assays. Triplicate cultures and assays were conducted. Columns are colonies from dilution series (10^{0}, 10^{-1}, and 10^{-2}) of each strain on representative plates. The LT column shows a representative colony of transformed AH109 strain plated on nonrestrictive synthetic dropout (SD) media (LT), confirming viability. Selective dropout media abbreviations and details of Y2H assays are described in Supplementary **Figure 4** and following manufacturer’s protocol. (**Left panel)** Protein interactions were assayed for growth on plates containing nonselective and restricted SD dropout media after 4 days. (**Right panel**) Protein interactions were monitored by secreted α-galactosidase (α-GAL) activity in liquid SD media -HLT plus 20 mM 3-AT media following 18 h of AH109 growth. Error bars are shown. (**D)** Schematic of TtPK1/OsSAPK2. See text and Figure 5.

Supplementary Figure 6. Co-immunoprecipitation (co-IP) Assays Confirm Dimerization. (A) Schematic of OsSCS1 with the “original prey” (14SSPSE, 12SSPSE) and deletion (12QPASQD, 12Δ) peptides; each designated by their final C-terminal residues. Detailed descriptions are in Figure 5C. **(B) Schematic of TtPK1** “original bait” (black bar) with 12 kinase catalytic subdomains (white roman numerals) and conserved kinase domain residues (above bar) and the aspartic acid-rich C-terminus. **(C) Cold reactions (cold rxn).** Non-radioactive rabbit reticulate lysate (RRL) reactions (cold rxn) were assembled to express the N-terminal T7-tagged proteins in pET28a (pET) in separate RRL reactions. An aliquot from each reaction was analyzed with SCS-PAGE and stained with Coumassie to confirm the quality and length of polypeptides. Shown is a representative gel with independent reactions: pET (lanes 1, 2); 14SSPSE (lanes 3-5); 12SSPSE (lane 7); and 12D (lane 8). Peptides produced were of the predicted size based on molecular markers (mw, lane 6). **(D) Hot reactions (**hot rxn). Radioactive RRL reactions were assembled to express the TtPK1 protein in pCITE (pC) with ^{35}S-methionine (NEN/Amersham). Cold and Hot RRL reactions were combined. For each combined reaction, anti-T7 peptide monoclonal antibody immobilized on protein A Sepharose beads (**) was added to co-immmunoprecitate (coIP) the “hot” protein. **(E) CoIP progress.** The supernatant and pellet after the beads were incubated with combined cold and hot RRL products. A phosphor-image of a representative gel of combined RRL products fractionated by SDS-PAGE and analyzed for quality and size. All lanes contained ^{35}S-methionine labeled TtPK1, except lane 4 with molecular markers (mw). Lanes are designated: 1-3 for the post-incubation supernatant; 5-7 for the post-incubation pellet; and 9-10 for the post first-wash (1x) pellet. Lanes 1 and 5 are designated with asterisks for reactions containing only the sepharose beads. Lanes 2 and 6 contained empty pET vector. Lanes 3, 7, and 10 contained 14SSPSE (14); and lane 8 contained 12SSPSE (12). **(F)** A phosphor-image of a representative gel for co-Ip assay. Each lane is an independent reaction**. Top of image** shows coIPs with ^{35}S-methionine labeled: TtPK1 (lanes 1-7), 14SSPSE (lane 8), empty pCITE vector (pC, lane 9), and catalytically dead TtPK1 (ΔG, lane 10).

Bottom of image shows coIPs precipitated with “cold” RRLs labeled: with asterisks for beads only (lane 1); with pET for empty vector (lane 2); with 14 for 14SSPSE (lanes 3, 6, 9, 10); with 12 for 12SSPSE (lane 4); with 12Δ for 12QPASQD (lane 5); or with TT for TtPK1 (lane 8). Lane 7 contained post-incubation TtPK1 pellet (as in lane7 of Supplementary Figure 6C) all other lanes were post wash1 pellets. Rainbow molecular markers (at a low concentration) were also in lane 7 for monitoring relative sizes on dried gel and phosphor-image. Lanes 8 and 9 contain ^{35}S-methionine-14SSPSE in pCITE or ^{35}S-methionine-empty pCITE, respectively, co-immunoprecipitated by cold T7-tagged TtPK1. **(H) A phosphor-image of a representative gel for replicate co-Ip experiment.**

Each lane is an independent coIPs reaction. All lanes are post-wash1x coIp pellets, except for lane 10, which is coIp input pellet. **Top of image** shows coIPs with ^{35}S-methionine labeled: TtPK1 (lanes 1-4, 10) and empty pCITE vector (pC, lanes 5-8). **Bottom of image** shows coIPs precipitated with “cold” RRLs labeled: with asterisks for beads only (lanes 1, 5); with pET for empty vector (lanes 2, 6); and with 14 for 14SSPSE (lanes 3, 4, 8, 10). Relative sizes were evaluated with molecular markers (mw, lane 9) and an aliquot of ^{35}S-met-TtPK1 RRL reaction (INPUT, lane 10) is indicated by arrow.

Supplementary Figure 7. **Controls for Bimolecular Fluorescence Complementation Expermiments** . Subcellular localization of **(A)** auto-fluoresence background control, **(B)** co-infiltration with empty-nYFP and empty-cYFP vectors, and **(C)** co-infiltration with empty-nYFP and cYFP-TtPK1 in *N. benthamiana* leaves transiently expressed from *Agrobacterium*-transformed constructs. Columns from left to right are images captured by bright-field, chloroplast auto-fluorescence, GFP, FM4-64 stain, DAPI-stain, and overlay. At least three independent infiltration experiments were conducted for each construct with 10-15 leaf sections observed and 5-10 images recorded.

Supplementary Figure 8. Subcellular localization of peptides transiently expressed in *N. benthamiana* leaves from *Agrobacteria N. benthamiana* -mediated constructs. Representative images show results of **(A)** N-terminal GFP-12SSPSE **(B)** at higher magnification; and **(C)** 3xNLS-GFP control and **(D)** at higher magnification. Columns from left to right are images captured by bright-field, chloroplast auto-fluorescence, GFP, FM4-64 stain, DAPI-stain, and overlay.

Supplementary Figure 9. ***OsSCS1*-hybridizing transcripts in Drying Seedlings**. **(A)** *OsSCS1* genomic locus (*Os03g145900*) with relative positions of five exons (rectangles) and four introns (lines) and its corresponding mRNA below (black arrow). Numbers above rectangles indicate nucleotide positions relative to start codon (hooked arrow); numbers within rectangles correspond to exon lengths (bp). The rectangle below is the predicted *OsSCS1* full-length coding region (~1179 bp). Relative nucleotide positions are indicated above the rectangles; relative amino acid positions are below and on the ends. Green and red boxes indicate relative locations of translational start and stop sites for the Y2H peptides: OsSCS1, 14SSPSE, and 12SSPSE. Features are as in **Figure 5**. The coding region (705 bp) of the randomly primed ^{32}P cDNA probe is underlined in red and designated 14LIE for **(B)** and **(C)**. RNA blots of relative accumulation of *OsSCS1*- and *rRNA*-hybridizing transcripts in total RNA extracted from **(B)** shoots (primary leaf/coleoptile tissues) of 14-d old rice seedlings after 16-h of drying (DRY) or hydration (H_2O). In (**B and C**), each lane had 15 μg of fractionated total RNA, which was stained with ethidium bromide to monitor size, quantity, and quality of total RNA. In **(B)** following transfer and hybridization, blots were exposed to the same phosphor-imaging screen for 3 and 60 hours. The ~1.4 kb length is consistent with the predicted full length size of the *OsSCS1* mRNA including UTRs. In (**B and C**), larger transcripts of ~3.5 kb were also present, likely corresponding to partially processed transcripts that still contain the large first intron. **(C)** RNA blot of relative accumulation of *OsSCS1*-, *TtPK1*-, or *rRNA*- hybridizing transcripts in total RNA extracted from shoot/coleoptile and crown tissue of 14-d old rice seedlings that were dehydrated for 0, 2, 4, and 8 hours. In **(C)**, the same blot was successively probed and gently stripped. An end-labeled ^{32}P rDNA primer of 26 nucleotides was used in all blots for probe controls with an exposure time of 5 minutes on a phosphor-imaging screen. **(D)** The *TtPK1* coding region used to prepare randomly primed ^{32}P cDNA probe is underlined in red. Note that in (C) larger

TtPK1-hybridizing transcripts of ~3.5 kb were also present, likely corresponding to partially processed kinase transcripts. Features are the same as described in Figure 5(A).

1.2 Supplementary Tables

Supplementary Table 1. List of primers

Supplementary Table 2. Taxa list for SnRK Phylogeny in Supplementary Figure 1 and Supplementary File 1.

Supplementary Table 3. Taxa list for SnRK2 Phylogeny in Figure 1, Supplementary Figures 2a-d, and Supplementary File 2.

Supplementary Table 4. Taxa list for SCS Phylogeny in Figures 2 and 3, Supplementary File 3.

Supplementary Table 5. Taxa list for SCS Phylogeny in Figure 4, Supplementary Figures 3a-c, Supplementary File 4.

1.3 Supplementary Files

Supplementary File 1. Multiple sequence alignment FASTA data format for SnRK Phylogeny in Supplementary Figure 1.

Supplementary File 2. Multiple sequence alignment FASTA data format for SnRK2 Phylogeny in Figure 1 and Supplementary Figures 2a-d.

Supplementary File 3. Multiple sequence alignment FASTA data format for Figures 2.

Supplementary File 4. Multiple sequence alignment FASTA data format for Figure 4 and Supplementary Figures 3a-c.
