# Supplementary figures and images for: Evolutionary Analysis of Snf1-Related Protein Kinase2 (SnRK2) and Calcium Sensor (SCS) Gene Lineages, and Dimerization of Rice Homologs, Suggest Deep Biochemical Conservation across Angiosperms
Source: Front Plant Sci. 2017 Apr 5;8:395. doi: 10.3389/fpls.2017.00395 (PMC5381359; doi:10.3389/fpls.2017.00395)

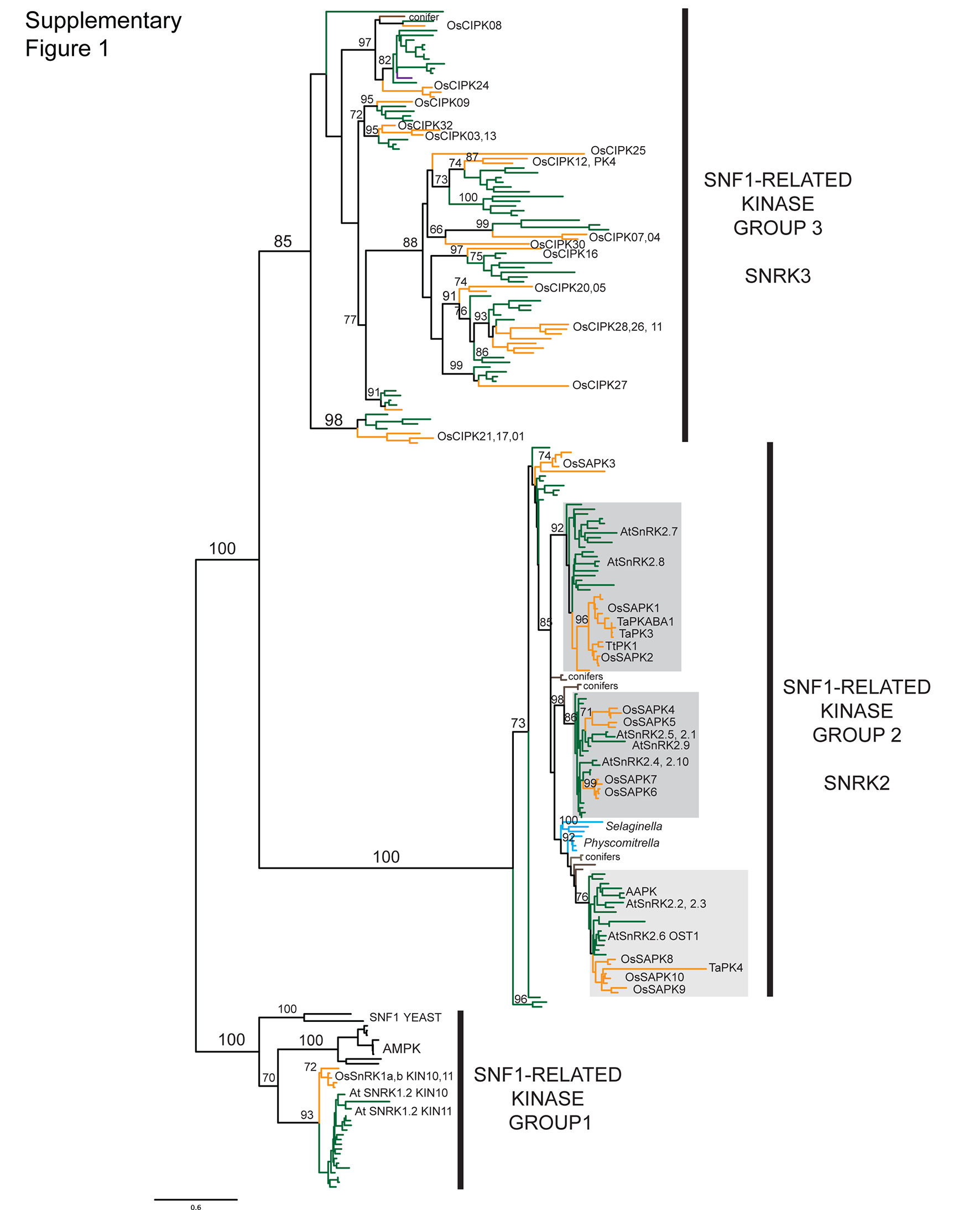

Supplement: Supplementary file 7 [file Image1.TIF]

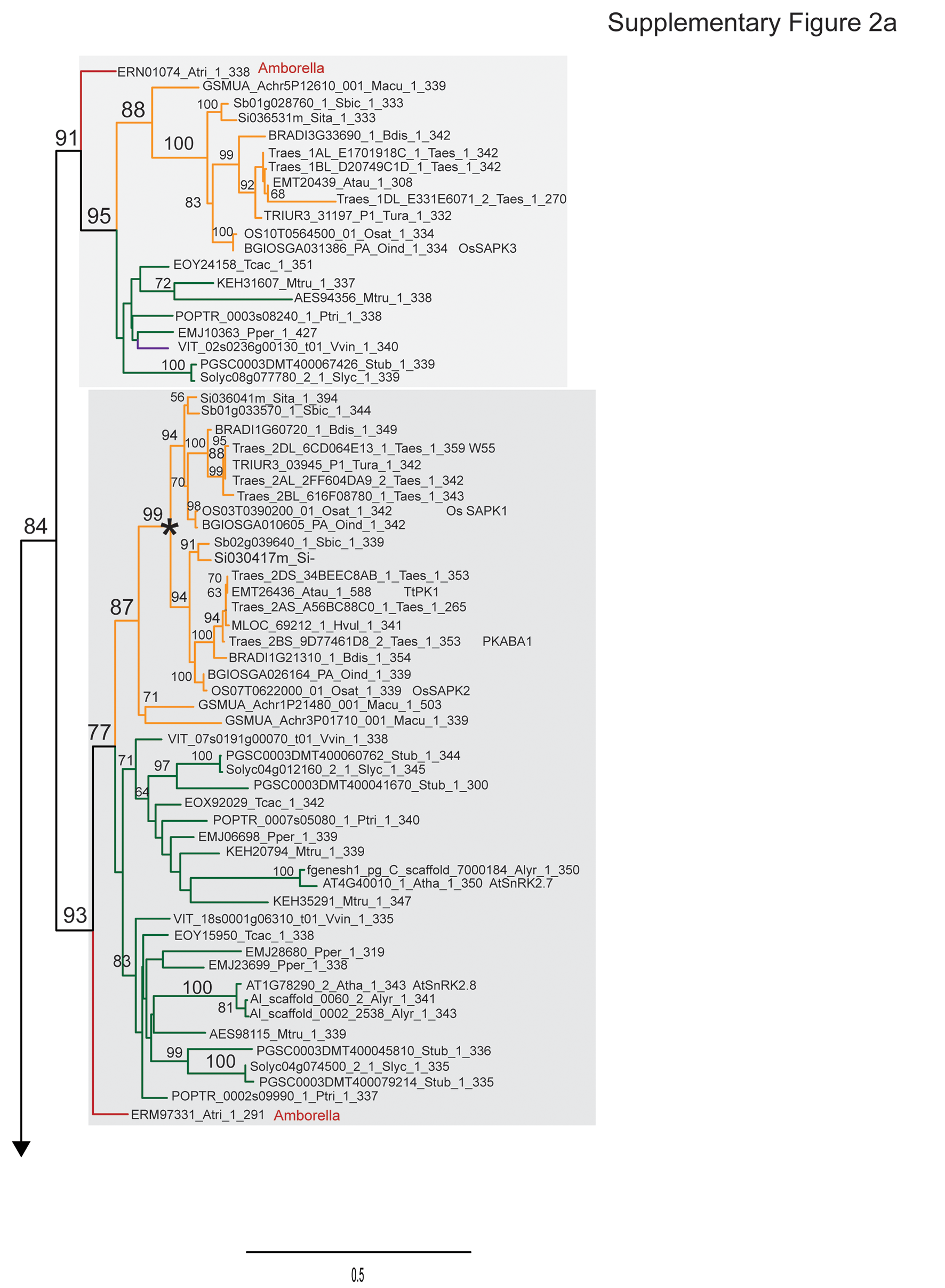

Supplement: Supplementary file 8 [file Image2a.tif]

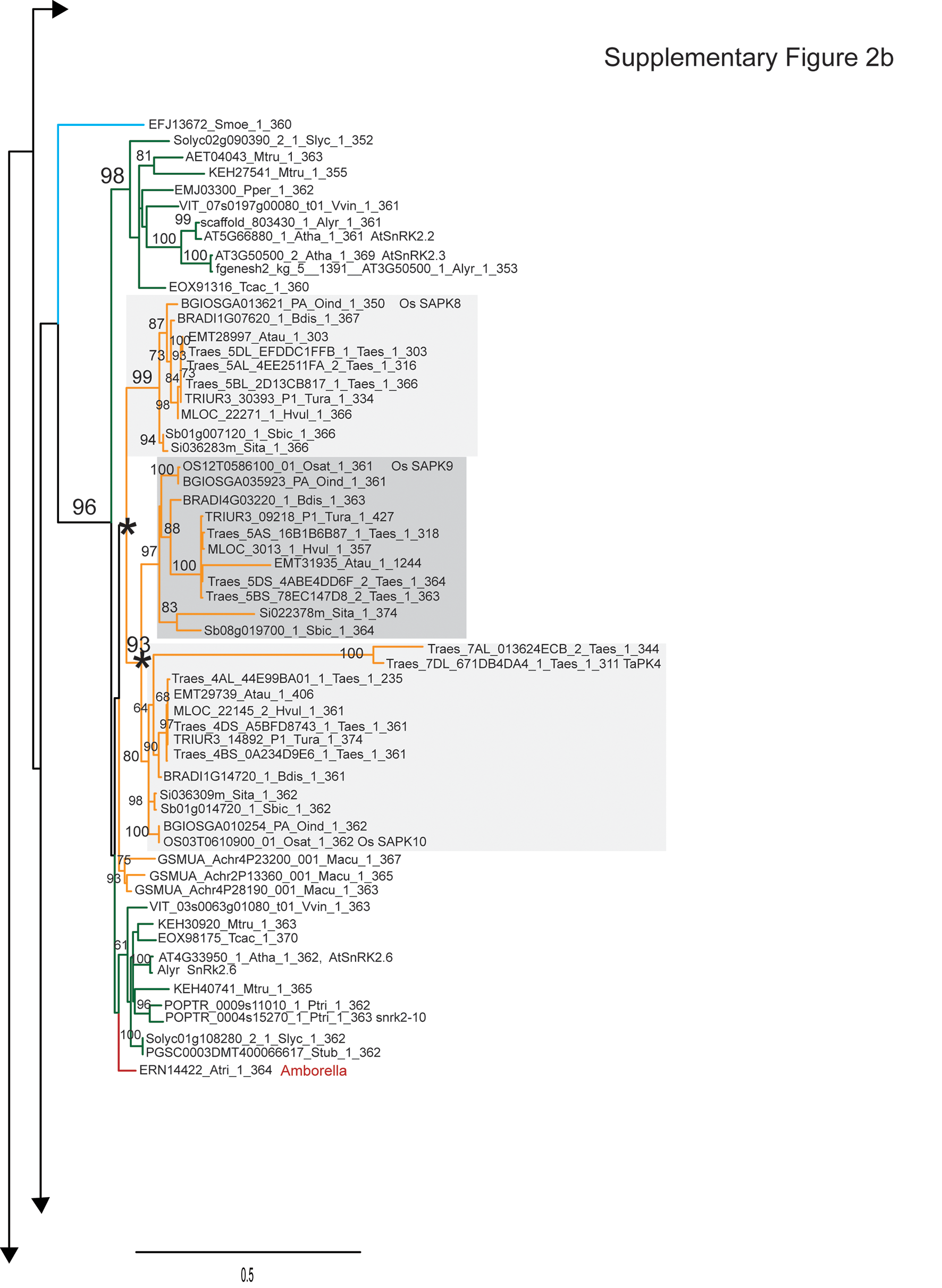

Supplement: Supplementary file 9 [file Image2b.tif]

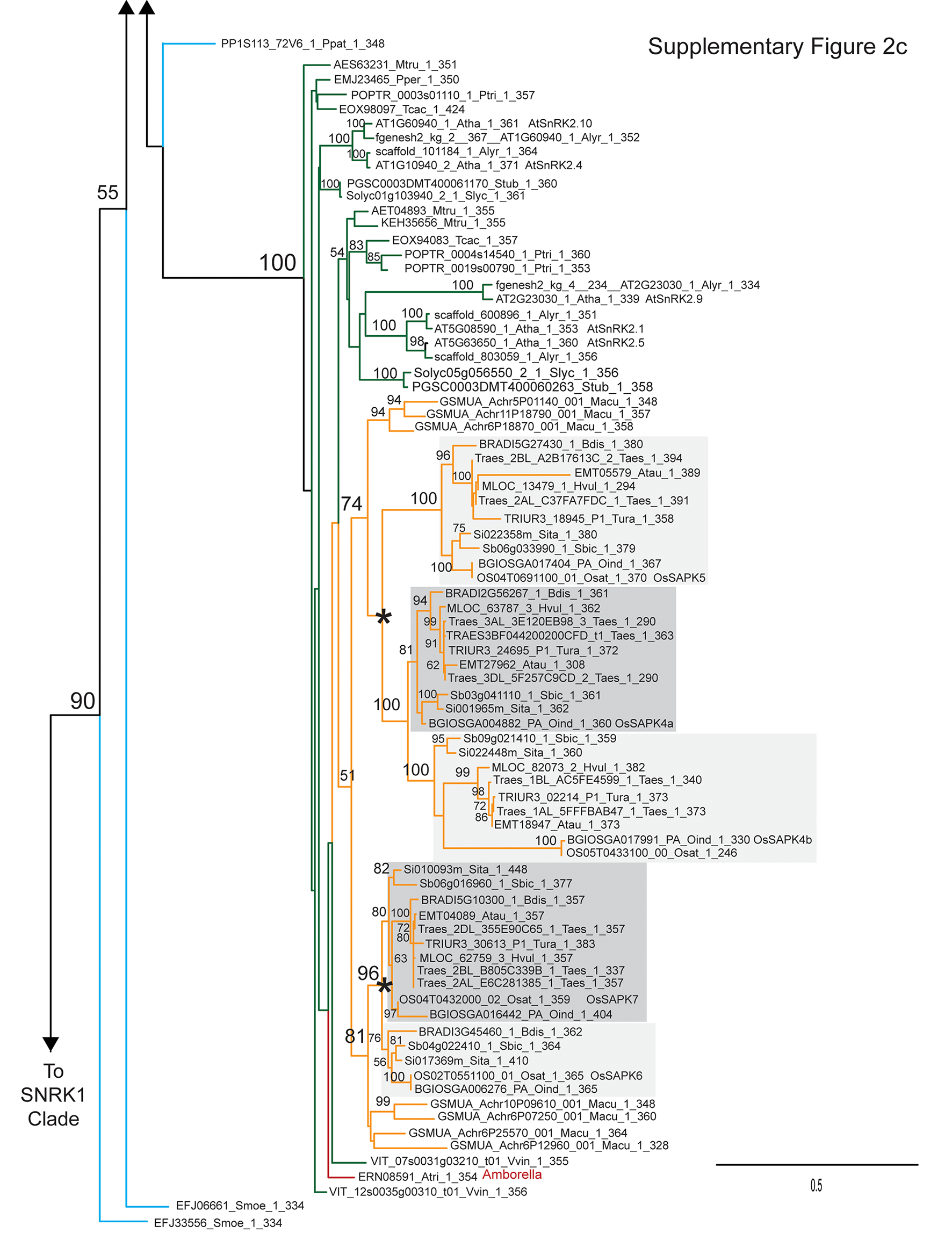

Supplement: Supplementary file 10 [file Image2c.tif]

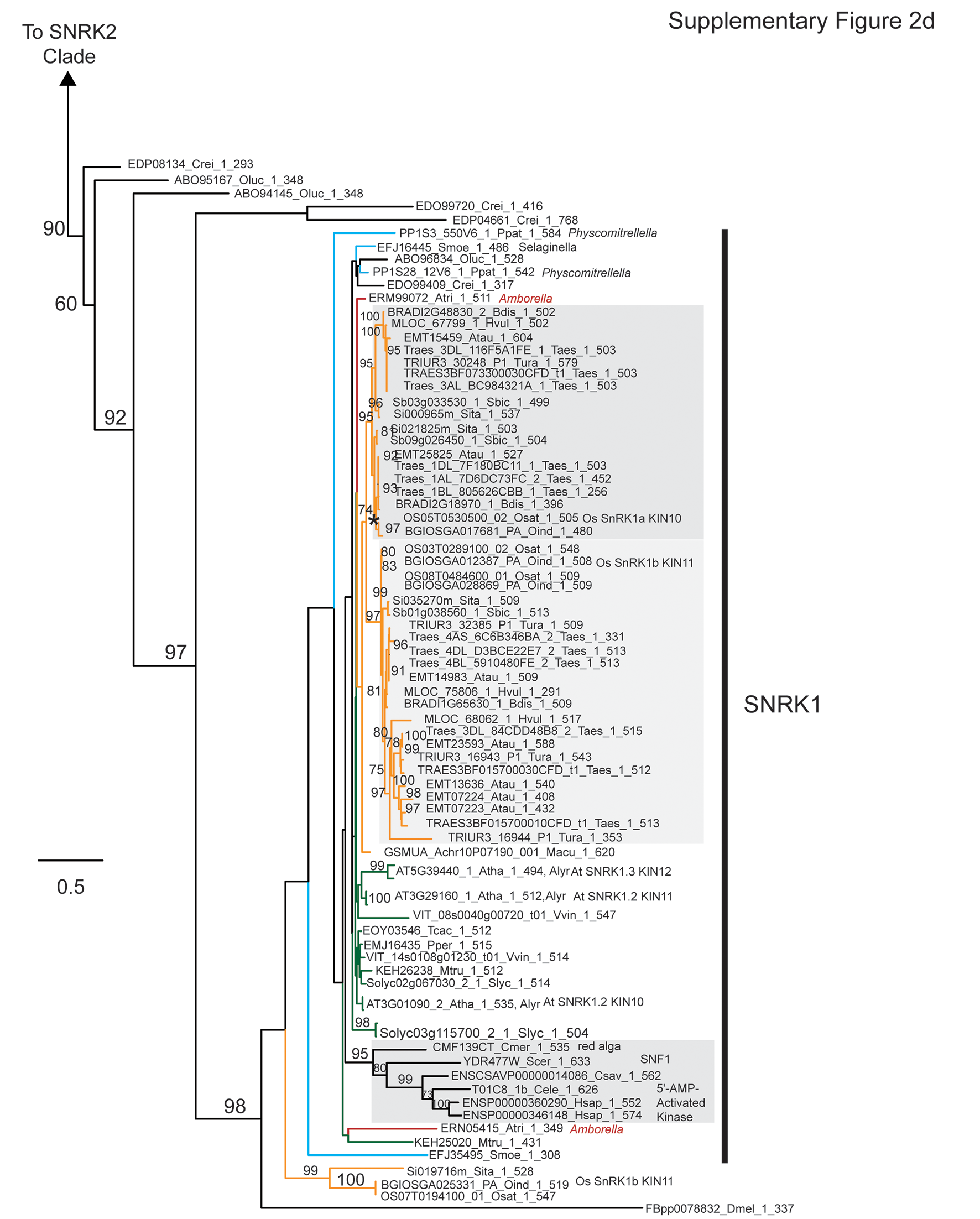

Supplement: Supplementary file 11 [file Image2d.tif]

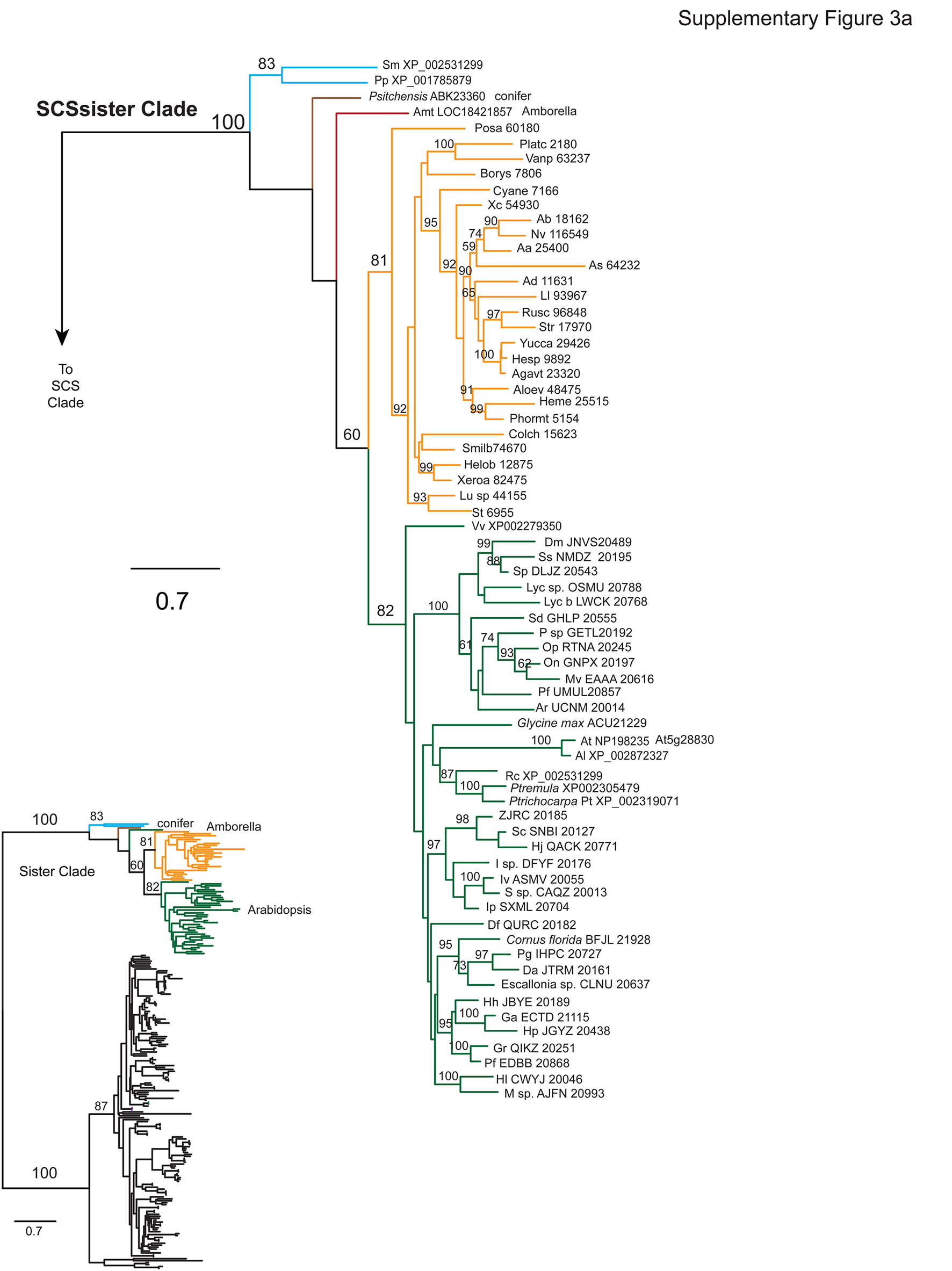

Supplement: Supplementary file 12 [file Image3a.tif]

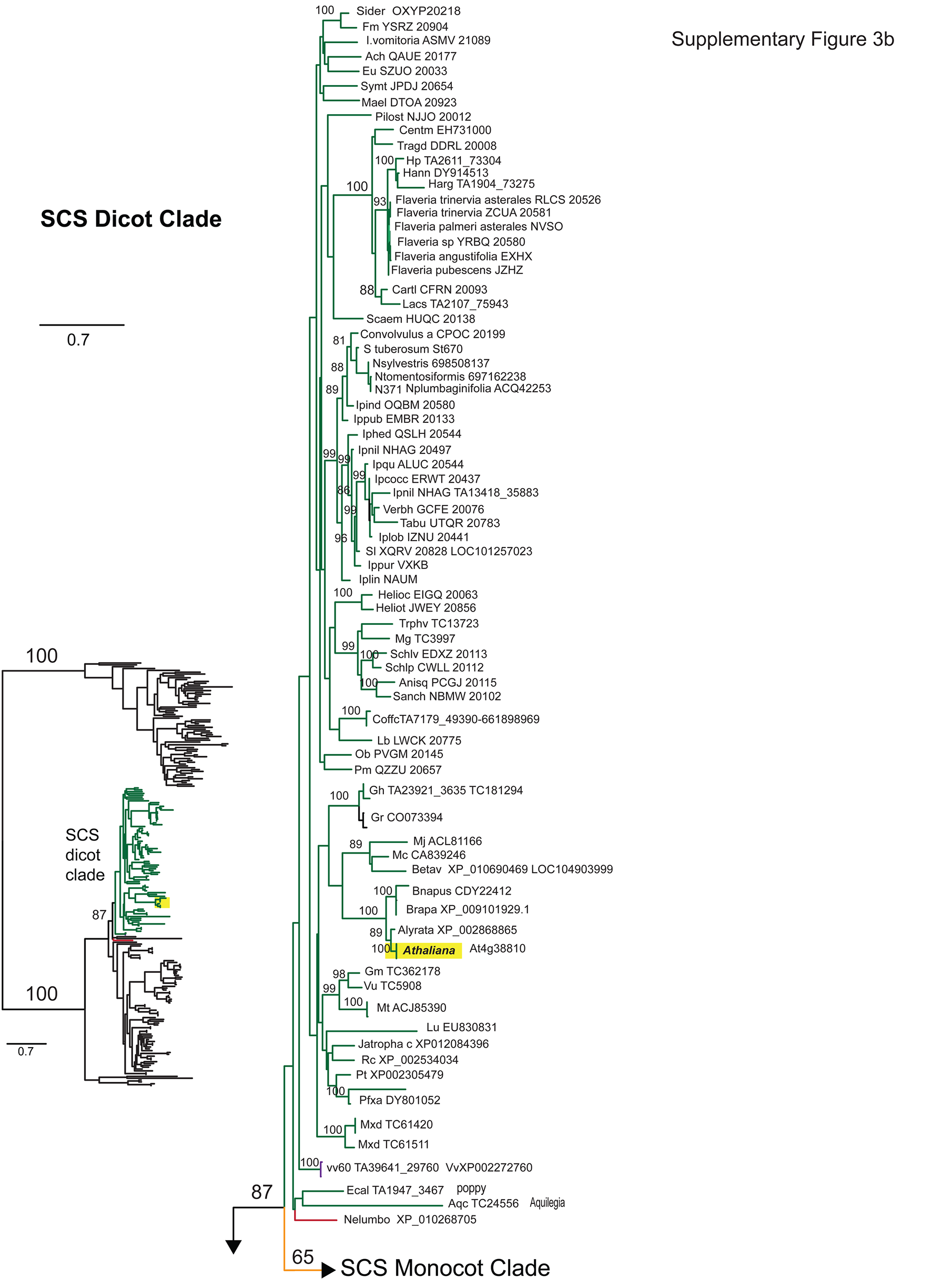

Supplement: Supplementary file 13 [file Image3b.tif]

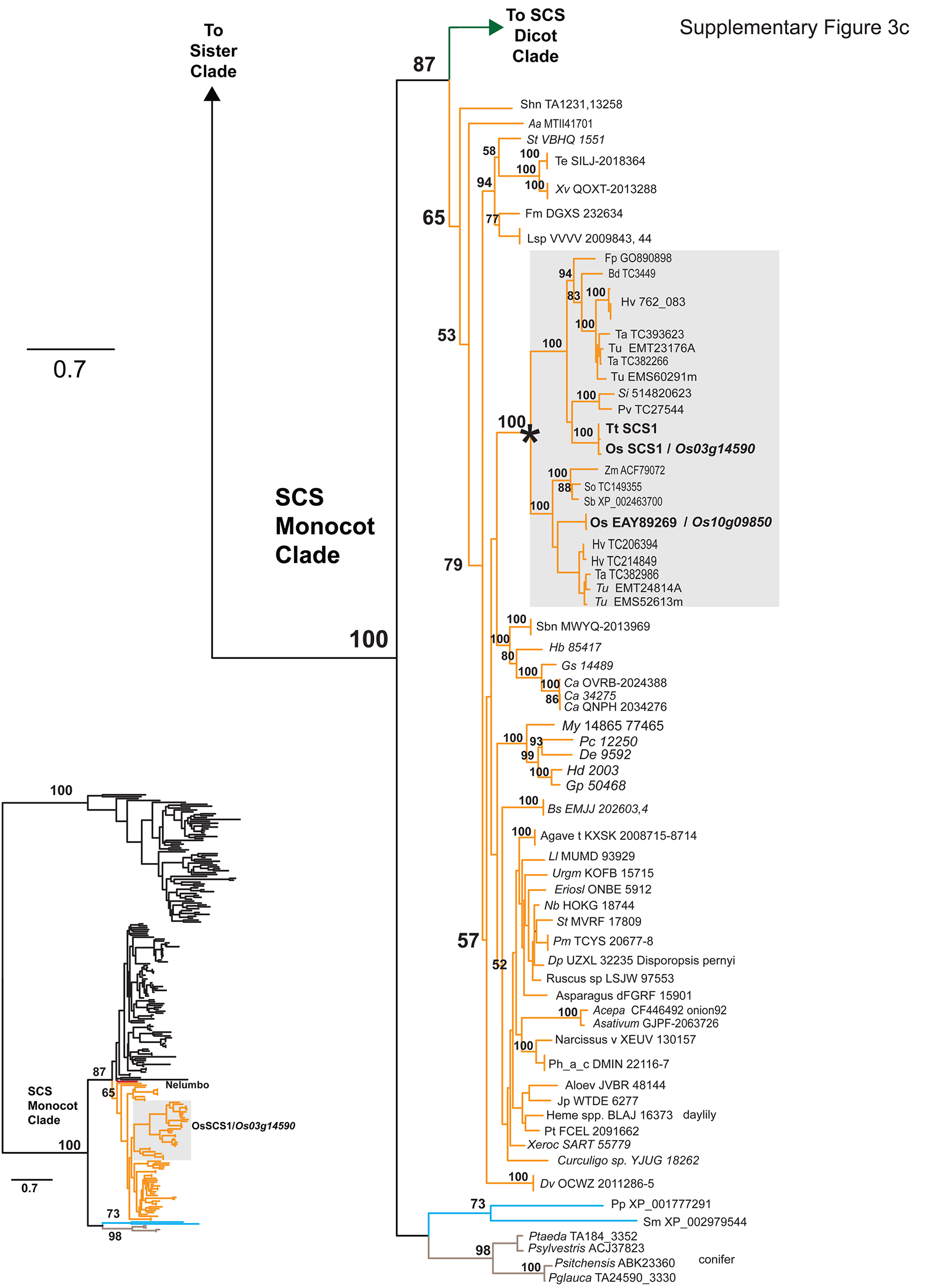

Supplement: Supplementary file 14 [file Image3c.tif]

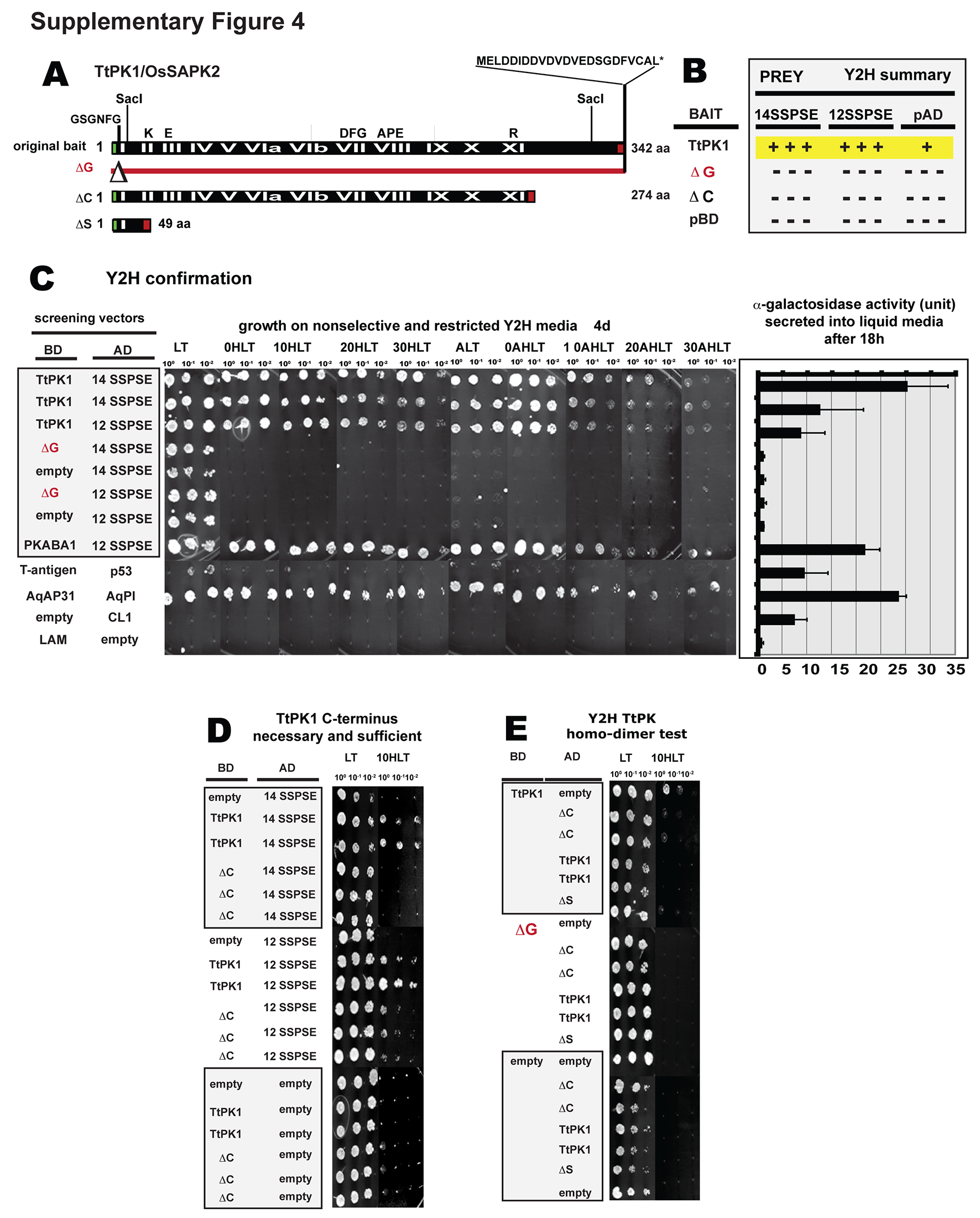

Supplement: Supplementary file 15 [file Image4.tif]

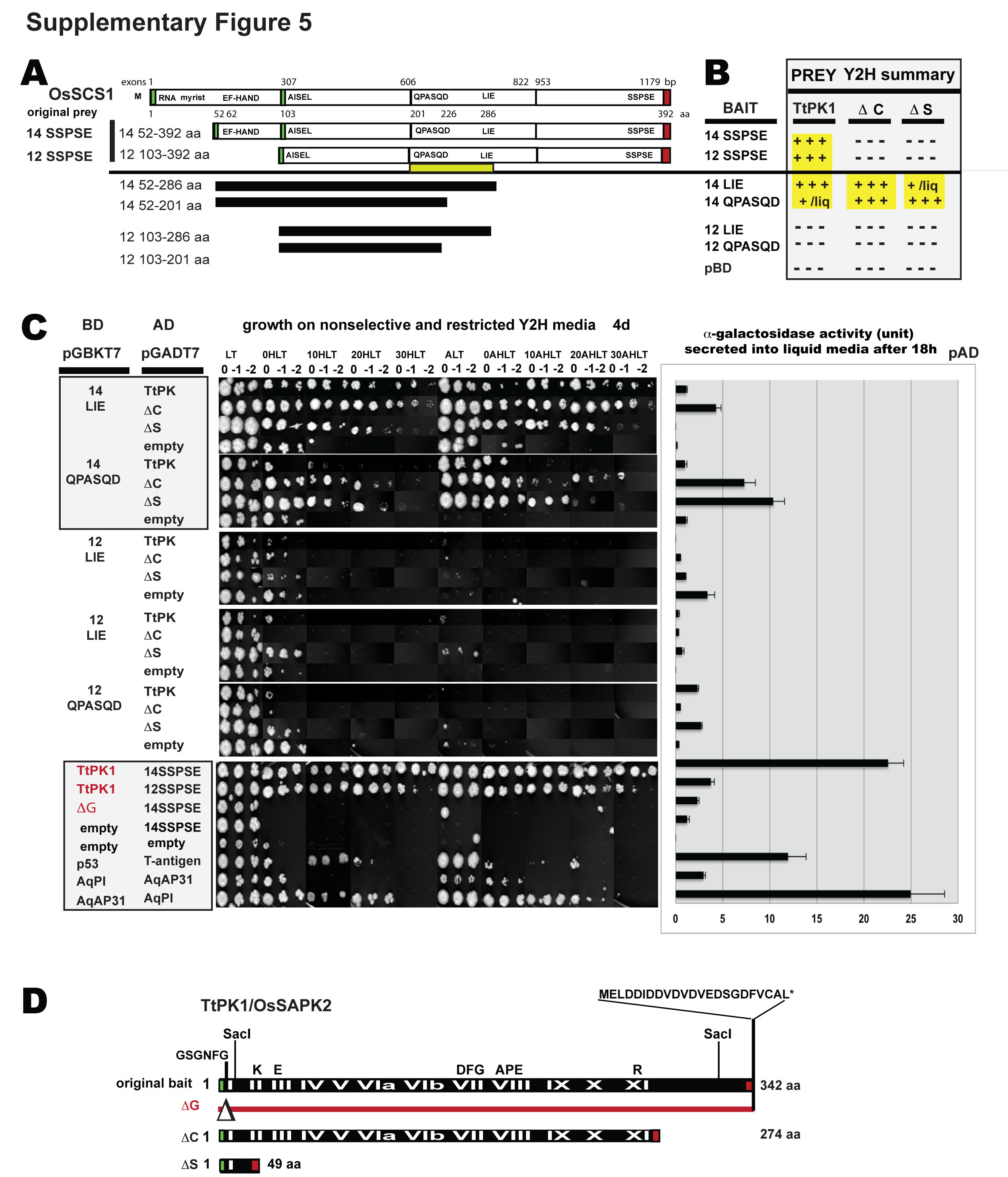

Supplement: Supplementary file 16 [file Image5.jpeg]

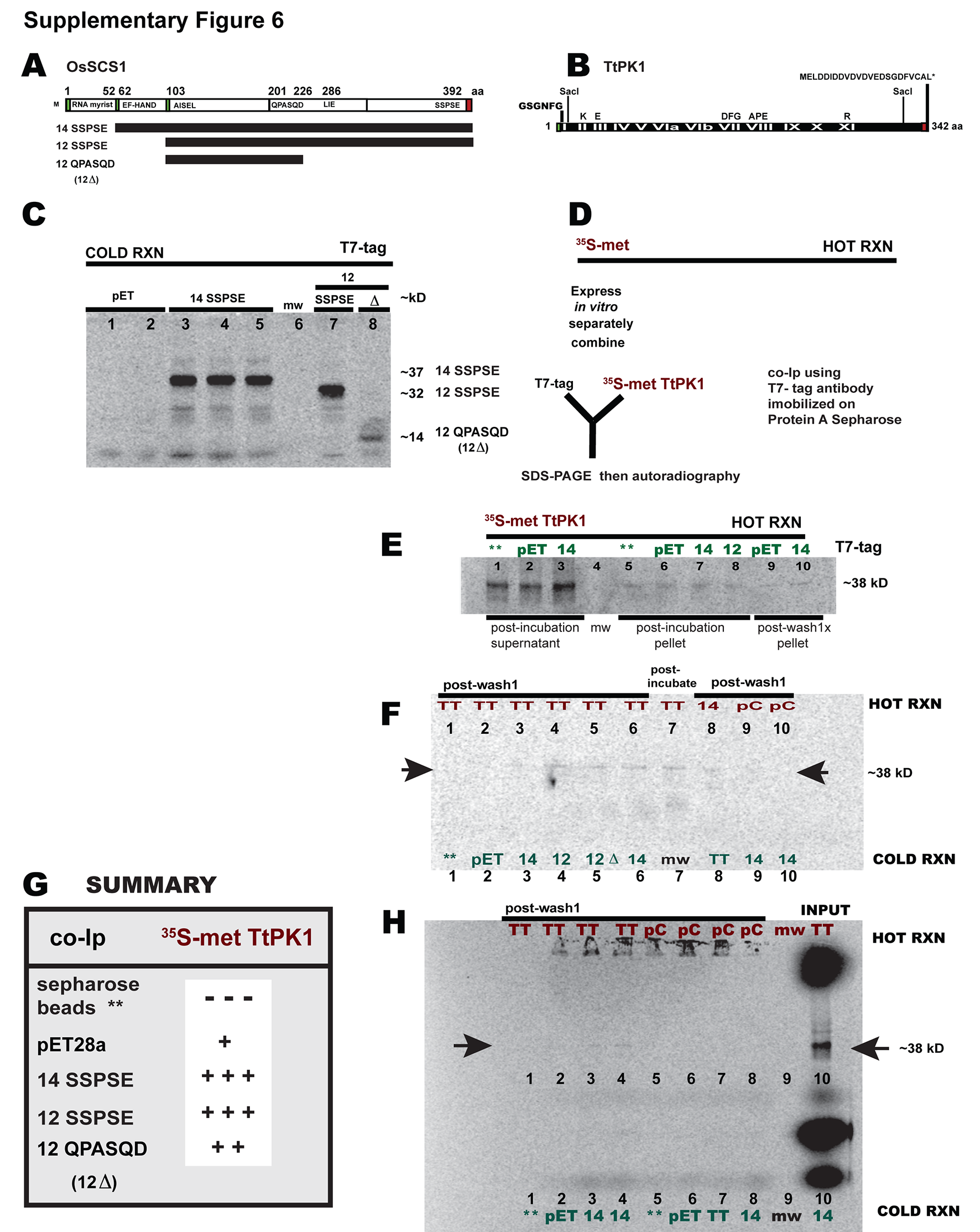

Supplement: Supplementary file 17 [file Image6.tif]

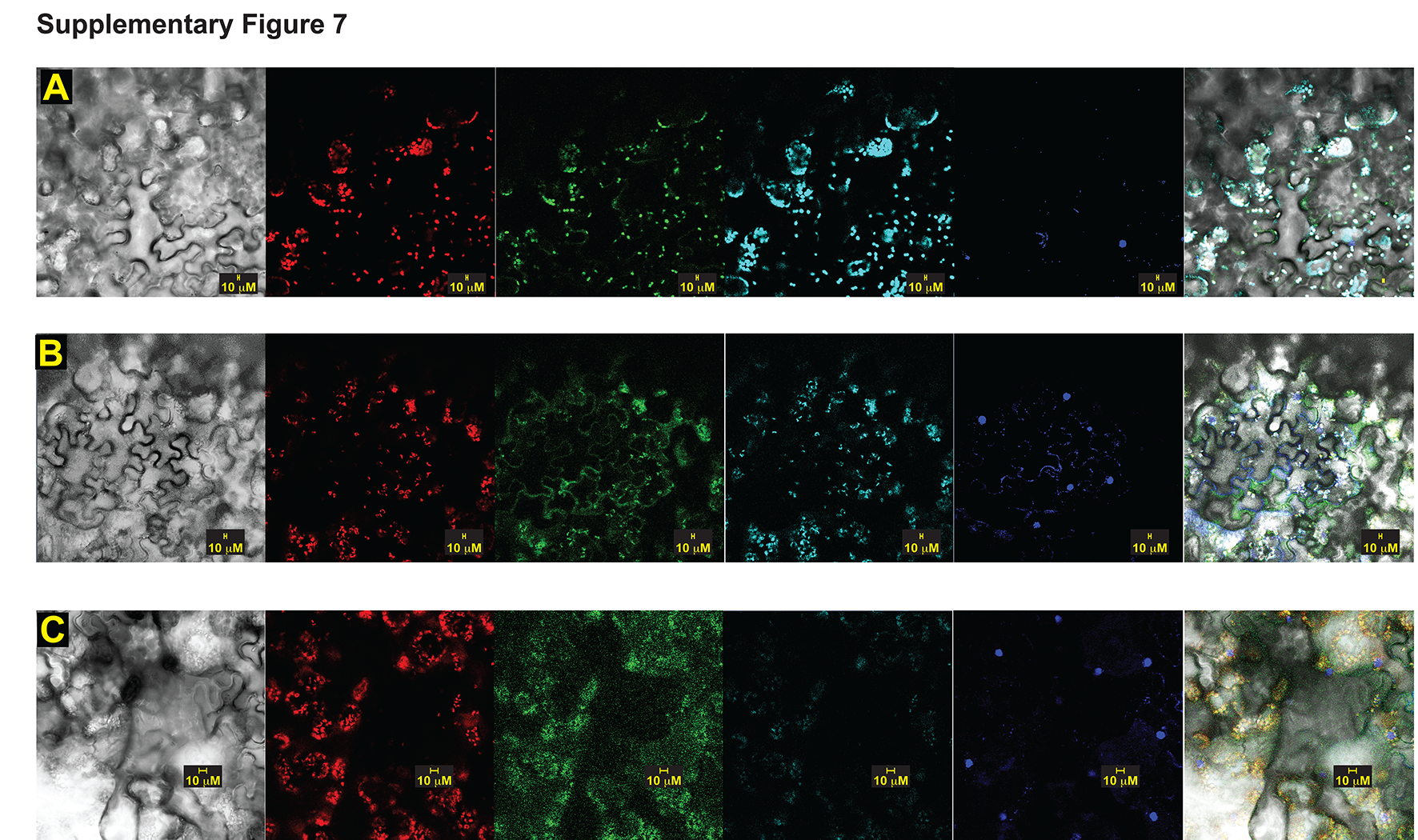

Supplement: Supplementary file 18 [file Image7.tif]

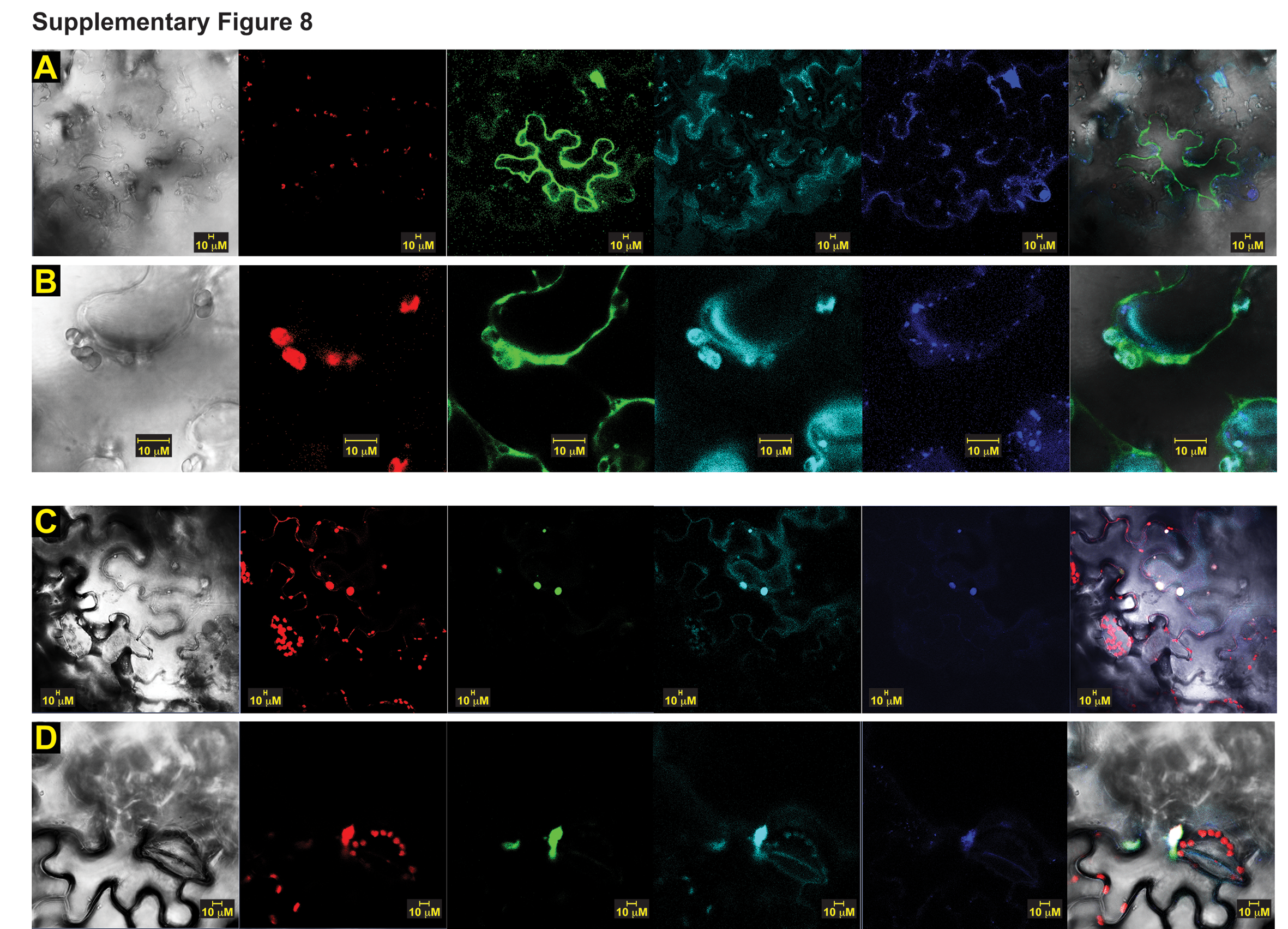

Supplement: Supplementary file 19 [file Image8.tif]

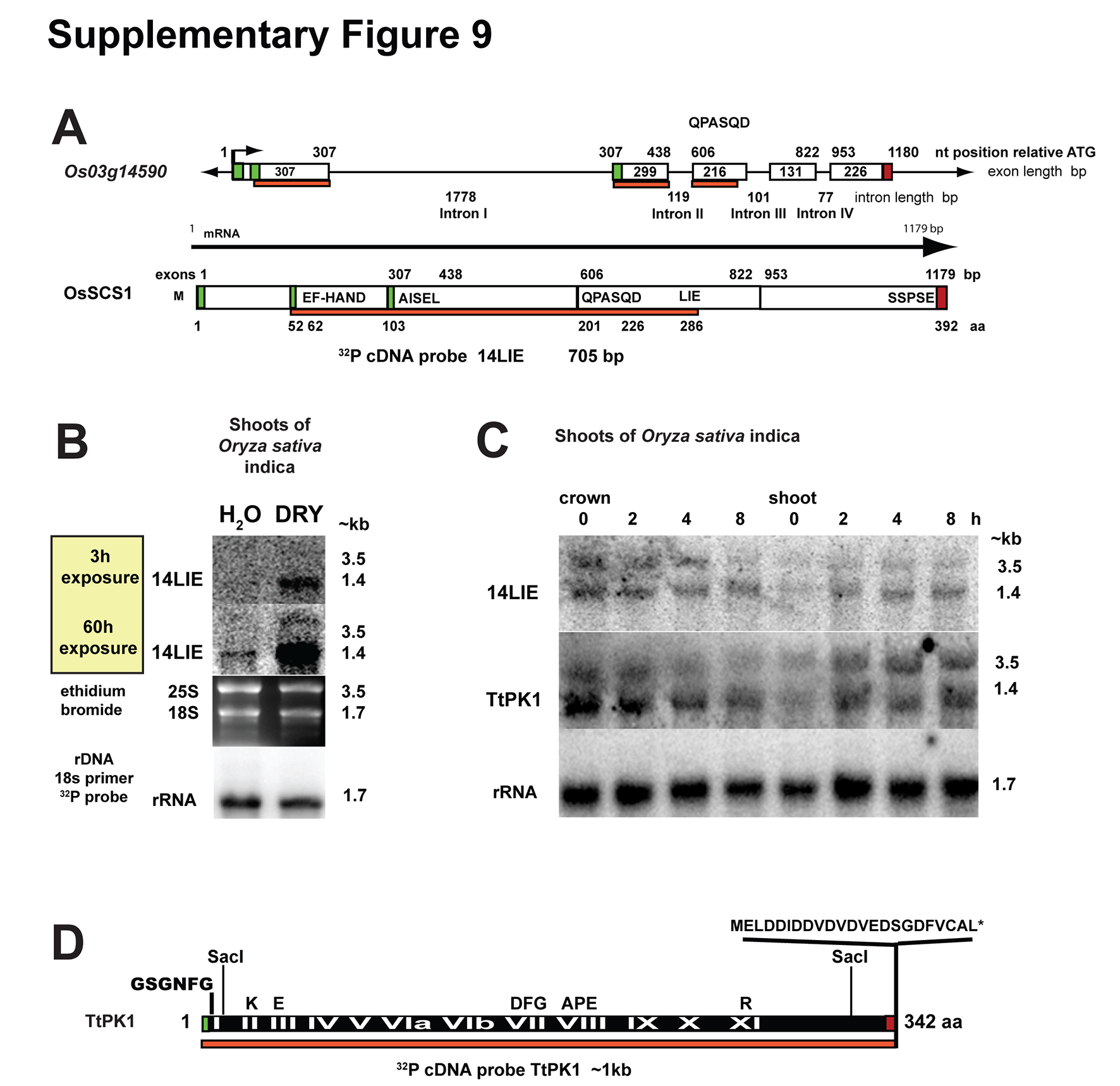

Supplement: Supplementary file 20 [file Image9.tif]
